# Directional virtual backbone based data aggregation scheme for Wireless Visual Sensor Networks

**DOI:** 10.1371/journal.pone.0196705

**Published:** 2018-05-15

**Authors:** Jing Zhang, Shi-jian Liu, Pei-Wei Tsai, Fu-min Zou, Xiao-rong Ji

**Affiliations:** 1 School of Information Science and Engineering, Fujian University of Technology, and Fujian Provincial Key Laboratory of Big Data Mining and Applications, Fuzhou, China; 2 Department of Computer Science and Software Engineering, Swinburne University of Technology, Hawthorn, Australia; Beijing University of Posts and Telecommunications, CHINA

## Abstract

Data gathering is a fundamental task in Wireless Visual Sensor Networks (WVSNs). Features of directional antennas and the visual data make WVSNs more complex than the conventional Wireless Sensor Network (WSN). The virtual backbone is a technique, which is capable of constructing clusters. The version associating with the aggregation operation is also referred to as the virtual backbone tree. In most of the existing literature, the main focus is on the efficiency brought by the construction of clusters that the existing methods neglect local-balance problems in general. To fill up this gap, Directional Virtual Backbone based Data Aggregation Scheme (DVBDAS) for the WVSNs is proposed in this paper. In addition, a measurement called the energy consumption density is proposed for evaluating the adequacy of results in the cluster-based construction problems. Moreover, the directional virtual backbone construction scheme is proposed by considering the local-balanced factor. Furthermore, the associated network coding mechanism is utilized to construct DVBDAS. Finally, both the theoretical analysis of the proposed DVBDAS and the simulations are given for evaluating the performance. The experimental results prove that the proposed DVBDAS achieves higher performance in terms of both the energy preservation and the network lifetime extension than the existing methods.

## Introduction

Wireless Sensor Networks (WSNs) consist of geographically distributed sensors that communicate with each other over wireless channels. A particular branch, which is equipped with the visual sensors for capturing target events through the video acquisition, called the Wireless Visual Sensor Network (WVSN) delivers the digital visual information via the wireless channels to the control unit further data analysis and decision making [1]. The WVSNs have a wide range of applications, such as visual security surveillance and visual wildlife monitoring [2]. The energy consumption pattern of the WVSN is very different from the conventional WSN. The reason is that the transmitted information in the conventional WSN are relatively small because it is generally digit data collected by the sensors with a single value or a few set of values. However, in the WVSN, the transmitted data is the video sequence, which is still much larger than the digit data even after the compression. Thus, the energy consumption of the WVSN is much larger than the conventional WSN [3]. In addition, the origin of inventing the WSN system is to create a wireless sensing and data transmission system under extreme environments such as the war zone, the large area of wasteland, or the disaster scene. The sensors are designed as disposable modules composed of a battery, a sensor unit, and a communication unit. It means that replacing or recharging the battery is impractical and sometimes impossible. Hence, designing the efficient data transmission protocols and routing algorithms are critical problems for the WVSNs to reduce the energy consumption in most applications.

In order the reduce the energy consumption caused by the data transmission, most of the WVSNs utilize the directional antenna to gain the equivalent transmission ability to the omnidirectional antenna but with lower energy consumption. With the directional sensors, a sensor node can sense partial sectors of a disk, which is centered at itself with the adjustable antenna [4]. The directional antenna [5] has a number of advantages over the omnidirectional antenna. By focusing energy only in the intended direction, the directional antenna is capable to increase the potential for spatial reuse and can provide longer transmission/reception distance than the omnidirectional antenna with the same amount of power consumption. The partially switched on sectors of a disk by the directional sensor node limits the toward direction of data forwarding but also reduces both the energy consumption for transmitting the data and the interference with other nodes. Different from the omnidirectional sensors, the discrete target coverage in the directional sensor network environment is determined by both the location and the orientation of the sensors. Thus, the complexity of routing and system optimization is significantly increased in the directional sensor networks such as the WVSNs. Since the characteristics of the directional sensor network and the omnidirectional sensor network is different, many existing methods used in the omnidirectional sensor network environments are not adaptable for using in the directional sensor network environments [6–9]. The need of intelligent schemes for capturing, processing, and transmitting visual data with the WVSNs with the resource-limited environment is undeniable. Answering to the need mentioned above, a Directional Virtual Backbone based Data Aggregation Scheme (DVBDAS) is proposed in this paper for solving the energy consumption optimization problem in the WVSNs. Our proposed scheme is capable to minimize the energy consumption but maximize the network lifetime by combining the clustering algorithm, the directional virtual backbone construction algorithm, and the network coding mechanism. The main contributions of this paper are listed as follows:

(1) A scheme called Directional Virtual Backbone based Data Aggregation Scheme (DVBDAS) for minimizing the power consumption and maximizing the network lifetime in the Wireless Visual Sensor Networks is proposed. The proposed scheme contains three procedures: the cluster construction, the directional virtual backboard generation, and the network coding mechanism deployment. In the DVBDAS structure, each cluster collects data from its own cluster. The cluster head forwards the collected data to the sink node through the route constructed by the virtual backbone data aggregation tree.

(2) A novel measurement called the energy consumption density (*D*_*ec*_) is defined in this paper for determining the cluster heads of all clusters by considering the load balance in the clusters. The defined *D*_*ec*_ provides answers to the crucial adapted cluster construction problem.

(3) An associated network coding and the directional control directional virtual backbone construction algorithm is proposed in this paper for building the energy preservation data aggregation tree in the WVSNs.

The rest of this paper is organized as follows: the related works of the topology control based data aggregation is briefly reviewed in section 2. Models and assumptions are defined in section 3. The proposed Directional Virtual Backbone based Data Aggregation Scheme is described in Section 4. The theoretical analysis and the simulation results are discussed in Section 5 and Section 6, respectively. Finally, the conclusion is made in Section 7.

## Related works

Topology control is a key issue, which must be addressed in WSNs. Several researchers have invested their thoughts and came up with many solutions to handle the topology control problem. However, the topology control method for the WVSNs is still vacant. In this section, several clustering-based topology control methods for WSNs are briefly reviewed. Although the WVSNs environment is different from the general WSNs, some similar concepts and principles can still be referable. The existing clustering-based topology control methods are divided into two categories based on the antenna types: the first one is the omnidirectional clustering, and the other one is the directional-antenna clustering. Details of these categories are given as follows.

### Omnidirectional-antenna category

The cluster-based topology control, which divides the network into several clusters, is known as one of the most efficient methods of the energy preservation in WSNs [10–19]. All sensors within a cluster only transmit data to their Cluster Head (CH), but the CHs in different clusters forward the aggregated data to the sink node. There are numerous well-known clustering protocols for WSNs been proposed. For example, Heinzelman et al. [10] propose the Low Energy Adaptive Clustering Hierarchy (LEACH) protocol, which is the first famous and efficient hierarchical routing protocol. In this protocol, CHs are selected in a fully distributed manner. LEACH-C is another routing protocol [11] following a centralized approach to select the CH using the base station and the location information of each sensor node. Younis and Fahamy propose a Hybrid Energy-Efficient Distributed clustering (HEED) protocol [12] in 2004. However, the process of choosing the CHs in HEED is operated in an iterative way. A new matrix that reflects the residual energy is fed as the reference data in HEED. Once the cluster members receiving multiple announcements with different CHs, the CH with the highest residual energy in the matrix is selected. Nayak and Devulapalli use a fuzzy inference engine to elect a super-CH (SCH) [13]. The SCH is selected among all CHs, of which can only send the information to the mobile base station, by choosing suitable fuzzy descriptors. Hosseini and Kahaei propose a cluster-based wireless sensor network massive multiple-input/multiple-output system [14], which is based on the Neyman-Pearson detection method. In their proposed method, sensors are divided into *k* clusters. In every cluster, a closed-form optimal solution is derived from the gain of each sensor. Marappan and Rodrigues propose an important parameter in the research area related to the design of routing protocols for Wireless Sensor Networks [15] in 2016. Javed presents an improved version of the LEACH protocol [16] in 2016. He utilizes the dynamic programming based intra cluster optimization technique with the Ant Colony Optimization, Voronoi Tessellation at inter clusters for energy efficient cluster head connection with the base station, and efficient coverage planning, respectively, to boost the performance of the LEACH protocol. Moreover, Liaqat et al. present a distance-based and low-energy adaptive clustering (DISCPLN) protocol [17] to streamline the green issue of efficient energy utilization in WSNs.

All cluster-based protocols mentioned above focus on the CHs selection. The discussion on the load balance is generally ignored in these methods and thus the lifetime of the CH with the highest load die much earlier than others and thus the lifetime of the whole network is shrunk. The overall power consumption of the system in the omnidirectional-antenna category is generally greater than those in the directional-antenna category because it requires larger energy to provide same transmission power in the omnidirectional range than in the partial sectors of a disk.

### Directional-antenna category

The virtual backbone construction technique can be found in the existing literature for finding the CHs. For instance, Imon et al. propose an efficient algorithm called the Randomized Switching for Maximizing Lifetime (RaSMaLai) [20] aiming at extending the lifetime of WSNs through load balancing. In their method, RaSMaLai randomly switches parts of the sensor nodes from their original paths to other paths with a lower load in the given data collection tree. Through several iterative cycles, the total power consumption drops along the found optimal paths. Kim et al. present a concept called the Average Backbone Path Length (ABPL) [21] in 2009. The ABPL utilizes the graph to describe the average routing path length and thus the path finding problem is equivalent to finding the minimum cost path in a graph. To avoid the energy being consumed on the unnecessary directions, Yang et al. define a Directional Connected Dominating Set (DCDS) [22] in the network composed of the directional antennas. Instead of focusing on the regular CDS-based visual backbone construction, DCDS focuses on selecting the sectors switched on forming a directional virtual backbone. The redundant energy consumption on the unnecessary directions can be removed because the antennas on the unnecessary directions will not be activated in the directional virtual backbone. On the other hand, Ding et al. propose the concept of the MOC-CDS [23], which is an algorithm considers the shortest path in the network. However, the constraints for composing the MOC-CDS tree is very strict and thus the CDS size is generally increased, greatly. To overcome this problem, Ding et al. propose another method called Virtual Backbone (*α* Minimum rOuting Cost Directional VB *α*-MOC-DVB) [24] in 2012. The MOC-DVB is capable to provide efficient broadcasting and routing abilities, which are the frequently used operations in the WSNs. Based on the knowledge of several classic distributed CDS approximation algorithm and the connected dominating set, Li et al. propose a new distributed CDS algorithm that considering the weight [25]. Zhang et al. put forward a directional neighbor discovery mechanism based on the model of optimization [26] in 2016. This mechanism adopts the Markov chain to model the random back-off state when beam aligns and the request frame is in transmit. A full bright view of the improvement is given in Table 1.

**Table 1:** Clustering algorithms.

Publication	Algorithm	Antenna Type
2000[10]	LEACH	Omnidirectional
2002[11]	LEACH-C	Omnidirectional
2004[12]	HEED	Omnidirectional
2016[13]	SCH	Omnidirectional
2016[14]	NP-based	Omnidirectional
2016[15]	routing protocol	Omnidirectional
2016[16]	improved-LEACH	Omnidirectional
2016[17]	DISCPLN	Omnidirectional
2015[20]	RasMaLai	Directional
2009[21]	ABPL	Directional
2008[22]	DCDS	Directional
2010[23]	MOC-CDS	Directional
2012[24]	*α*-MOC-CDS	Directional
2010[25]	weight-based	Directional
2016[26]	optimization	Directional

Based on the truth revealed in the existing literature, it is known that the directional-antenna network is more efficient than the omnidirectional-antenna network in term of energy preservation. In addition, the load balance factor is nearly all off the list of the consideration in the existing literature. This paper aims to create a directional virtual backbone construction algorithm, which considers the load balance for the directional-antenna network of the WVSNs.

## Problem statement and assumptions

The energy consumption minimization for the data collection in WVSNs is an NP-complete problem. To come up with the solution, the aim of this paper is to establish the corresponding scheme for the WVSNs with the clustering operation and the network coding mechanism. To construct the directional virtual backbone, both the load balance and the characteristic of the directional antenna are taken into the consideration. In this section, the definitions and assumptions for the proposed scheme including models of the directional antenna, the network, and the energy consumption are given as follows.

### Directional antenna model

**Fig 1:**
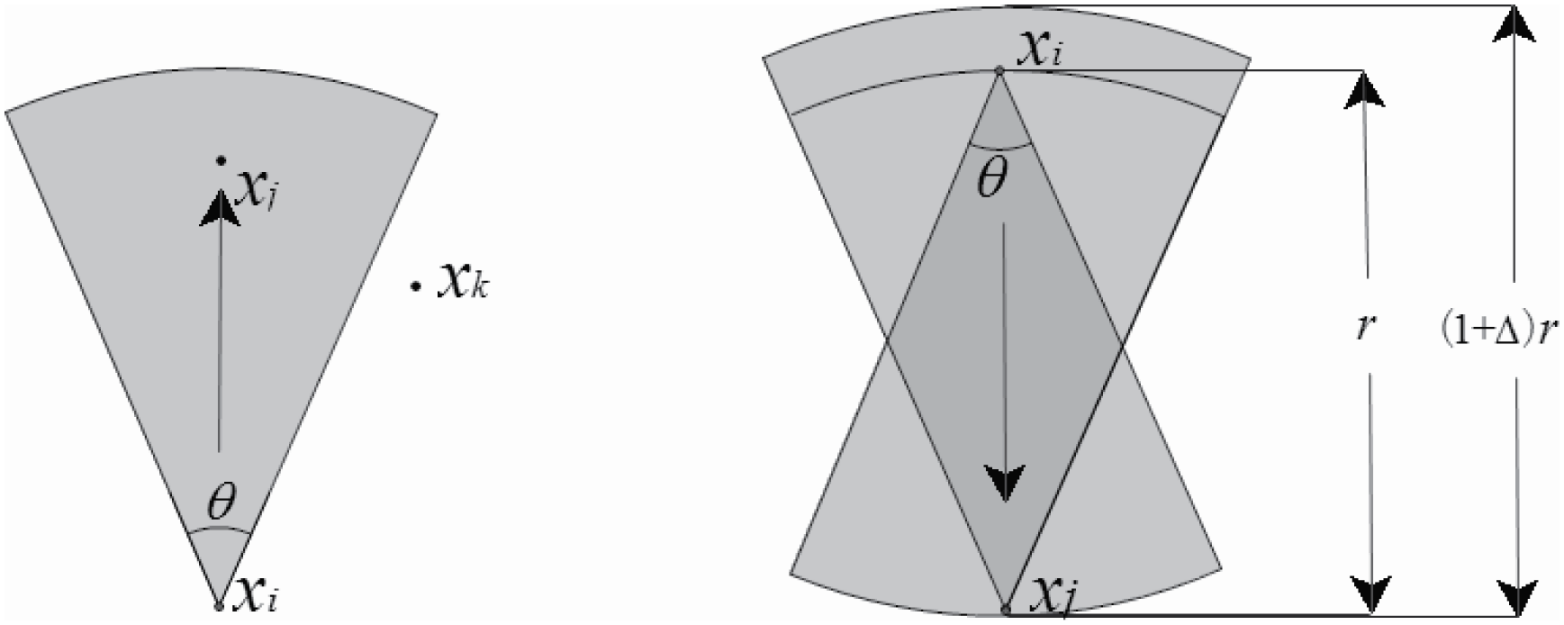
Directional antenna model.

There are numerous directional antenna models for the wireless network can be found in the existing literature [27]. In this paper, the directional antenna is presented as a circular sector with the angle *θ* and the radius equals to the transmission/reception range *r*. The directional antenna model is shown in Fig 1. The directional antenna gain is within the specific angle *θ*, which is the beam-width of the antenna. The gain outside the beam-width is assumed to be zero. It is defined that *x*_*i*_ can transmit a packet to *x*_*j*_, but *x*_*k*_ cannot receive the packet since it is outside of the beam-width of *x*_*i*_. It is generally well-known that modeling a real directional antenna is complex, the directional antenna pattern consists of a main-lobe which is the direction of the maximum radiation/reception and several smaller back-lobes arising due to inefficiencies in the antenna design. As shown in Fig 1, the transmission/reception range may be (1 + △)*r*. For the simplicity, a simple directional antenna is used in our model, that is to say the variable range △ is ignored in this paper.

**Fig 2:**
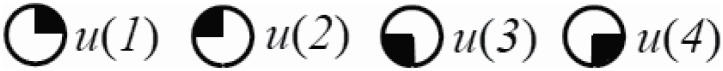
Directional antenna for node *u* with $\theta =\frac{\pi }{4}$.

The proposed scheme, which can be adjusted as required, in this paper is suitable for any kind of directional antenna environment. However, to describe the operation simply, we assume $\theta =\frac{\pi }{4}$. As shown in Fig 2, there are four sectors with different directional antennas. It means that the transmission range of each node is divided into four uniform sectors. The *i*^*th*^ direction of node *u* is denoted as *u*(*i*). Furthermore, every antenna in the network is adjustable. Each sensor can define its antenna in any desired direction. In some particular cases, all sectors of an antenna can be used at the same time to gain the same ability as the omnidirectional antenna.

### Network model

The graph theory is used to model the communication behavior of the WVSN. The visual sensor nodes are randomly deployed in the network field as the same as in the conventional WSN for monitoring the environment via the camera. The transmission range *r*_*i*_ of a sensor node *v*_*i*_ is variable along with the change of the transmission power. In addition, the collected data is sent to the sink node by the multi-hop operation. The network is modeled by a connected and directed graph *D* = (*V*, *E*), where *V* and *E* represent the node set and the link set in *D*, respectively, when the link between any pair of the cluster heads is directional. For each node pairs *u*_*i*_, *u*_*j*_ ∈ *V*, there exists an edge (*u*_*i*_, *u*_*j*_) in *D* if and only if *u*_*i*_ is in $u_{j}^{\prime }s$ transmission range in the network there is no obstacle preventing radio wave transmission between *u*_*i*_ and *u*_*j*_.

Furthermore, the cluster construction technique is also used in this paper. A cluster covers a circular area centered at its CH *o* with the sensing radius *r*_*o*_. When the visual data is collected by the cluster members, the collected data is forwarded to the cluster head. An efficient virtual backbone is constructed among all CHs and is rooted at the sink node. The CHs encode the received data as the message by the network coding technique. The message will be forwarded to the sink node through the virtual backbone. To complete the virtual backbone structure, some assumptions are made in this section.

**Definition 1**. (Directional Neighbor) Consider a node *u*. The set of nodes covered by the *i*^*th*^ directional antenna of *u* is represented by *N*(*u*(*i*)), *N*(*u*(*i*)) = {*v* ∣ (*v*, *u*) ∈ *E*}, which is called the open neighbor set of *u*(*i*). *N*[*u*(*i*)] = *N*(*u*(*i*)) ∪ {*u*} is called the closed neighbor set of *u*(*i*). $N\left(u\right)={⋃}_{i=1}^{4}N\left(u\left(i\right)\right)$

**Definition 2**. (Cluster) [28] The cluster-based network consists of many non-overlapping clusters. In a cluster *C*_*i*_, there is only one cluster head *CH*_*i*_, which is responsible for establishing the cluster and asking the sensor nodes to join this cluster. Other modes in the cluster are called the Cluster Members (*CMs*).

**Definition 3**. (Network Coding) [29] In the network coding process, coding nodes merges the data flow by the mathematical operations. The encoded message is compressed in the encoding process and thus reduces the size of forwarded message remarkably and further reduces the energy consumption of the data transfer. The number of messages encoded by a coding node in each transmission is called the encoding number.

In this paper, the CHs are all equipped with the ability to be the coding nodes. Since the CH is taking the responsibility of collecting data of its cluster and encoding the received data before transmission, the energy consumption of the CH is naturally larger than the ordinary nodes. The energy consumption model is introduced in the next subsection.

### Energy consumption model

The energy consumption is directly related to the size of the transmitted packet *k* and the transmission distance *d* between the transmitter and the receiver nodes. We use the multi-path fading channel models as in [10, 30], which are shown in the Formulas (1) and (2):

$$\begin{array}{c} E_{Tx}\left(k,d\right)=E_{elec}*k+E_{fs}*k*d^{2}\left(d<d_{tr}\right) \end{array}$$ (1)

$$\begin{array}{c} E_{Tx}\left(k,d\right)=E_{elec}*k+E_{mp}*k*d^{4}\left(d\geq d_{tr}\right) \end{array}$$ (2)

where *E*_*elec*_(*nJ*/*bit*) is the electronics energy, *E*_*fs*_(*pJ*/*bit*/*m*^2^) and *E*_*mp*_(*pJ*/*bit*/*m*^2^) are the energy amplifier coefficients that depends on the distance to the receiver, and *d*_*tr*_ is a distance threshold between the transmitter.

The consumed energy for receiving a packet can be formulated by Formula (3):

$$\begin{array}{c} E_{Rx}=E_{elec}*k \end{array}$$ (3)

**Definition 4**. (Data Generation Rate) The data generation rate $R_{i}^{g}$ is defined as the amount of data generated by sensor *v*_*i*_ in a data collection round.

**Definition 5**. (Data Reception Rate) The data reception rate $R_{CH_{i}}^{r}$ of *CH*_*i*_ is defined as the amount of data *CH*_*i*_ receives from its children in a data collection round.

**Definition 6**. (Transmission Rate) The transmission rate $R_{CH_{i}}^{t}$ of *CH*_*i*_ is the amount of data transmitted by the cluster head *CH*_*i*_ in a data collection round as well.

**Definition 7**. (Energy Loss Rate) The energy loss rate of the cluster head *CH*_*i*_ is given by:

$$\begin{array}{c} E_{CH_{i}}=R_{CH_{i}}^{t}E_{Tx}+R_{CH_{i}}^{r}E_{Rx}. \end{array}$$ (4)

**Definition 8**. (Energy Consumption Density) The energy consumption density *D*_*e*_*CH*_*i*___ is a metric for the load balance, which is the rate between the intra-cluster energy consumption *E*_*CH*_*i*__ and the area of the region the cluster head covered *S*_*CH*_*i*__.

**Definition 9**. (Node lifetime) The load *L*_*CH*_*i*__ of a cluster head *CH*_*i*_ is defined as the ratio of *E*_*CH*_*i*__ to the energy it maintains *EM*_*CH*_*i*__. The lifetime of a cluster head is given by:

$$\begin{array}{c} T_{CH_{i}}=EM_{CH_{i}}/E_{CH_{i}}=1/L_{CH_{i}}. \end{array}$$ (5)

**Definition 10**. (Network Lifetime) The lifetime of a network is defined as the lifetime of the virtual backbone lifetime time *T*_*VB*_, which is the minimum lifetime of all nodes in *VB*. Formally,

$$\begin{array}{c} T_{VB}=min\left\{T_{CH_{i}}|CH_{i}\in VB\right\}. \end{array}$$ (6)

## Network coding and clustering-based data aggregation scheme

Clustering and network coding is essential to remove the redundancy and reduce the size of the data. In the proposed Directional Virtual Backbone based Data Aggregation Scheme (DVBDAS) for the WVSNs, the CHs collect data from their own clusters and encode the data into message packets before forwarding the packets to the sink node via the virtual backbone data aggregation tree. The DVBDAS is composed of three procedures:

(1) Cluster construction: Through the cluster construction process, the whole network coverage area can be treated as the union of sets composed of clusters.

(2) Directional virtual backboard construction: Constructing an efficient directional virtual backbone for transmitting the visual data back to the sink node.

(3) Network coding by the CHs.

### Cluster construction

The cluster construction aims to the whole network coverage area into several clusters. It can be divided into 4 steps: the network configuration, the cluster centroid initialization, the load balance-based CH selection, and the CH update.

**Step 1 (Network Configuration)**: Since multi-hop strategy is used in the data forwarding mechanism, the energy consumption of CHs nearby the sink node would be higher than the others because more packets need to be forwarded by them. In order to balance the regional energy consumption, the regional autonomy method is used in this algorithm. Every region has a CH selection probability, that is to say sensors in this region become a CH with probability *p*_*i*_, which is determined by the distance to the sink node and the network density.

Once sensor nodes are deployed in the network field, they have the information about their relative coordinates. Driven by a hand-shake mechanism, the sensor node sends packet containing its sensing range *r*_*i*_, the sensor *ID*, the union set of the neighborhoods *N*(*u*_*i*_), and the directional antenna to the Sink *Dir*_*s*_ in the network as the “hello message” to its neighbors at the beginning. The hello message is defined as:

$$\begin{array}{c} hello\;message=\{r_{i},\;ID,N\left(u_{i}\right),Dir_{s}\}. \end{array}$$ (7)

**Step 2 (Cluster Centroid Initialization)**: To initialize the clustering algorithm, sensor nodes from the candidate list are randomly selected to be the initial CHs. A sensor node, of which the current energy *E*_*i*_ is equal to or greater than a predefined threshold *E*_*TH*_ is eligible to be selected as the CH. Thus, all sensor nodes can be classified into two possible cases listed as follows.

Case 1: (*E*_*i*_ < *E*_*Th*_): The current energy level of the sensor node is lower than the threshold and thus it can not be selected as the CH. The node is marked as a normal node and is excluded from the candidate list.

Case 2: (*E*_*i*_ ≥ *E*_*Th*_): The sensor node has sufficient energy and it is collected in the candidate list. The probability of choosing this sensor node to be the CH is denoted by *p*_*i*_. If a node is selected to be the CH, it broadcasts a packet called “candidate message” to the others with the content of the Maintained Energy *EM*_*i*_, a union set of its neighborhoods *N*(*u*_*i*_), and its *ID*. The candidate message can be defined by Formula (8):

$$\begin{array}{c} CH\;candidate\;message=\{E_{i},N\left(u_{i}\right),\;ID\}. \end{array}$$ (8)

**Step 3 (Load balance-based CHs Selection)**: In the previous step, the CHs are initially selected based on the energy levels. To optimize the selected CHs, the load balance needs to be taken into account for selecting the CHs because the ideal case is that the energy consumption of all CHs can be unified. According to Definition 7, the energy consumption of a CH with its members are physically located far away from it could be higher than a CH with more members but located nearby it. It implies that considering only the density of the cluster member is not sufficient. Thus, we define the energy consumption density *D*_*ec*_ to present the rate between the intra-cluster energy consumption and the region the cluster head covered defined in Definition 8 and use it as the factor for the consideration. The load of CHs would be more balanced when *D*_*ec*_ is involved in the decision. The optimal number of the CHs is defined as follows:

$$\begin{array}{c} N_{opt}=\alpha_{1}\frac{1}{D_{ec}}+\alpha_{2}EM_{i}+\alpha_{3}\frac{N(u_{i})}{S_{area}}. \end{array}$$ (9)

where *N*_*opt*_ denotes the optimal number of the CHs, *α*_1_, *α*_2_ and *α*_3_ (*α*_1_ + *α*_2_ + *α*_3_ = 1) are the influence factors that can be put with different weights based on the needs.

The *N*_*opt*_ is emphasized by the fact that the energy consumption is well distributed over the clusters. *N*_*opt*_ is higher when the remained energy of the CH is higher. If there is a neighbor node *v* whose optimal number is higher than *u*, then *v* will be selected as a cluster head and the CH message will be broadcasted by it.

The sensor node is merged into a cluster once it receives the CH candidate message. If a node receives more than one CH candidate message (such as that the node *v*’s neighbor CH *N*_*ch*_[*v*] > 1), it is merged into the cluster, of which the CH is physically the nearest to it. In order to estimate the efficiency of the algorithm, a data aggregation level network based load balance factor is assumed. In the data aggregation application, “funneling effect” may be appeared in the several-to-one data flow network. It means that the load of nodes nearby the sink node is greater than the others. Hence, the distance between the sink node and the CH is used as the weight of the original load. The weighted load is defined as *L*_*i*_ = *l*_*i*_ × *d*_*i*_. Assume there are *n* CHs, of which the weighted loads are denoted by *L*_1_, *L*_2_, …, *L*_*n*_ and all weighted loads are greater than 0. Since the data aggregation is a constant, we have *L*_1_ + *L*_2_ + … + *L*_*n*_ = *C*, where *C* is the data aggregation constant. According to the in-equation theory, there is

$$\begin{array}{c} \sigma =\frac{{\left(\sum_{i=1}^{n}L_{i}\right)}^{2}}{n\sum_{i=1}^{n}L_{i}^{2}}\leq 1, \end{array}$$ (10)

where *σ* is the load balance factor denoting the load balance level. The load balance is much greater when *σ* is approaching to 1. Especially, when *L*_1_ = *L*_2_ = … = *L*_*n*_, the left side of the in-equation will achieve the maximum value. In other words, the load balance is achieved. Based on these three steps, the cluster construction algorithm is shown in Algorithm 1.

**Algorithm 1**: Cluster Construction

1 with probability *p*_*i*_, each node broadcasts *hello* *message* = {*r*_*i*_, *ID*, *N*(*u*_*i*_), *Dir*_*s*_};

2 **if** (*E*_*i*_ ≤ *E*_*Th*_) **then**

3  who wants to become a candidate cluster head will broadcasts the ‘candidate cluster head’ message: *CH* *candidata* *message* = {*E*_*i*_, *N*(*u*_*i*_), *ID*};

4 **end**

5 its neighbor whose optimal number *N*_*opt*_ is higher than the candidate cluster head, it will be the cluster head;

6 broadcasts the ‘cluster head’ message;

7 calculates the load balance factor;

8 **if** (*σ* < *TR*_*σ*_) **then**

9  cluster head reselect;

10 **end**

**Fig 3:**
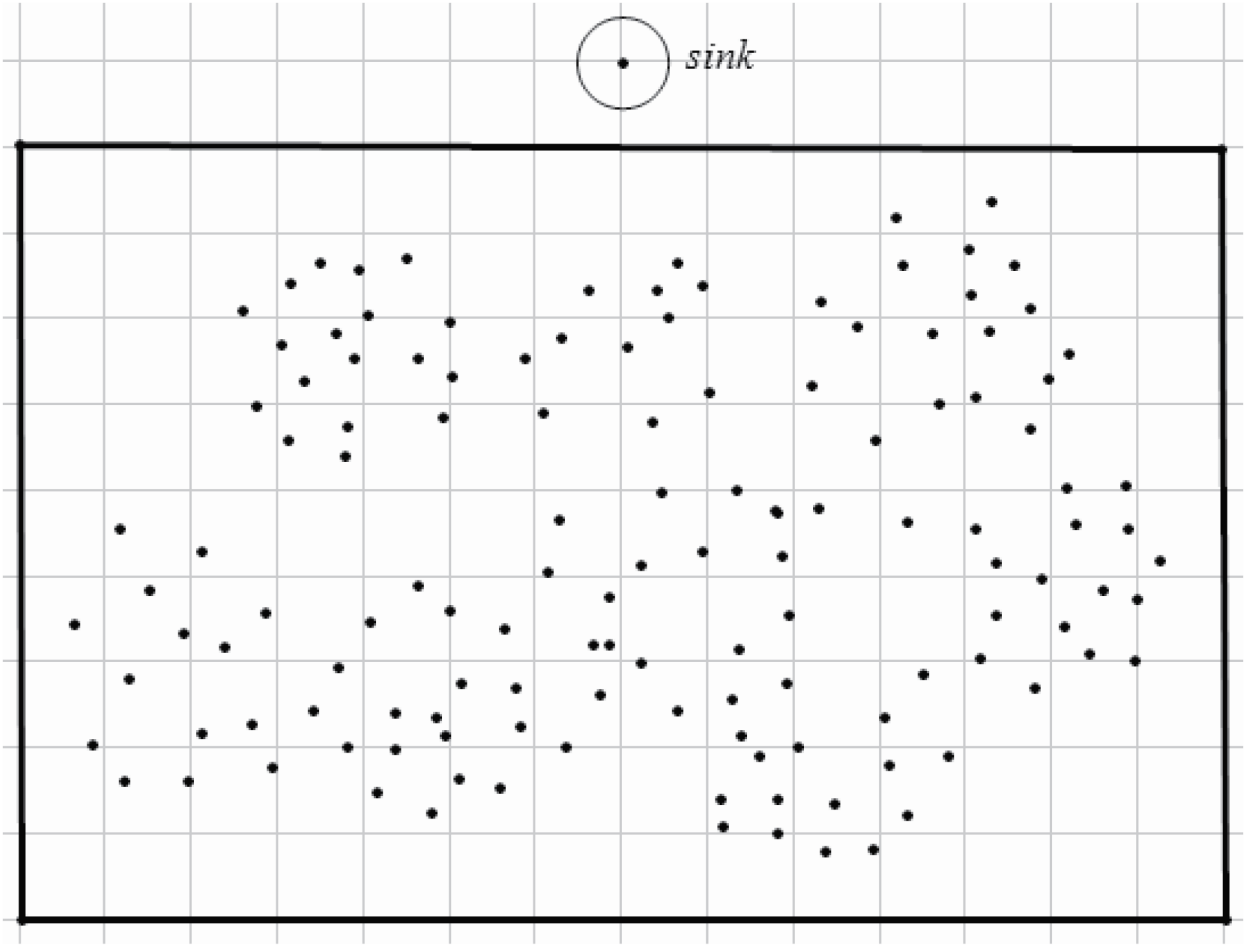
An origin wireless visual sensor network.

**Fig 4:**
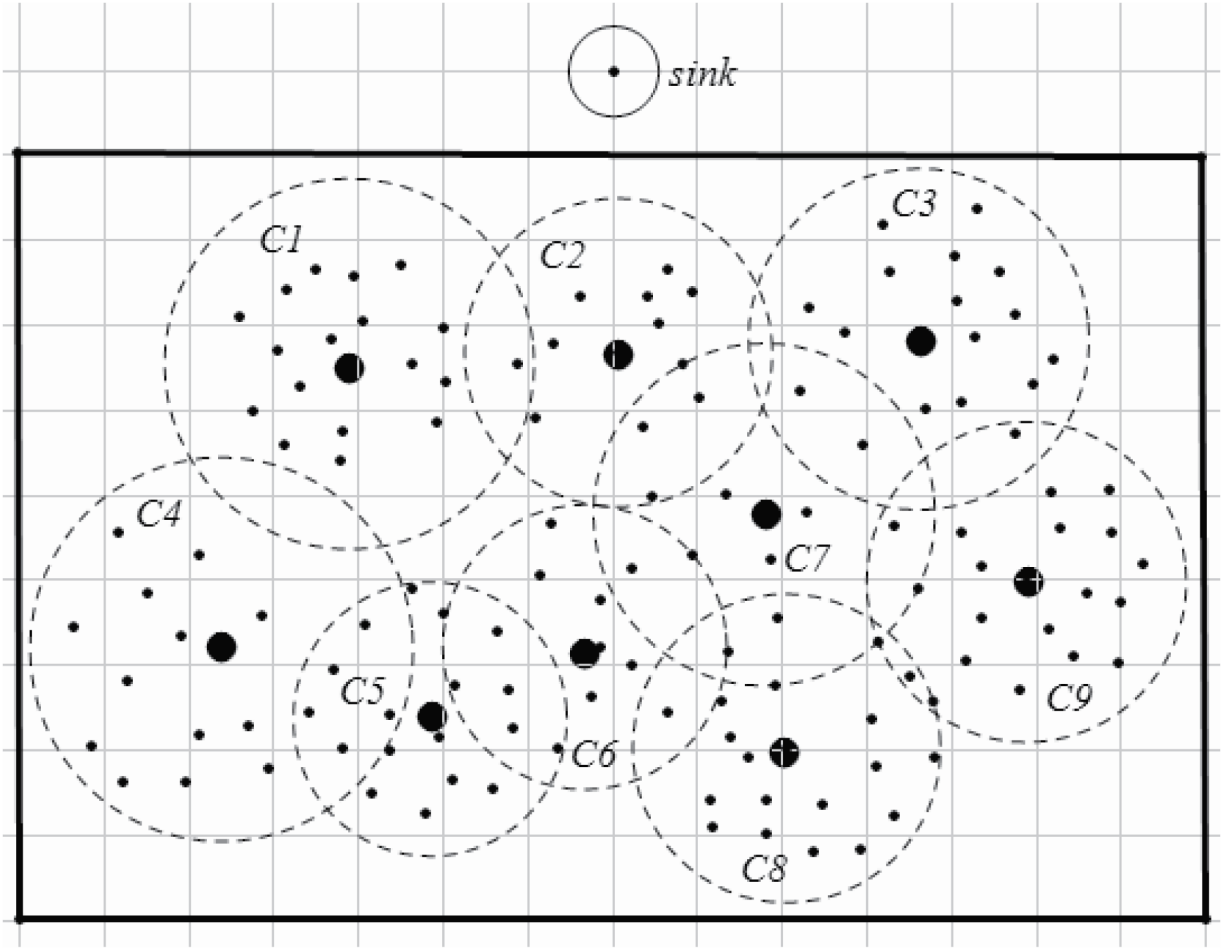
Cluster construction and cluster head selection.

11 those who receive the cluster head broadcast message can decided to be the cluster head’s cluster member;

An example is shown in Figs 3 and 4. Fig 3 shows an original network, visual sensors are disposed in the area interested. In this example, the interested area is a rectangle. The sink node is in the front of the rectangle. After step 2 (lines 1-4 in Algorithm 1.), each sensor is assigned to a specific CH. However, the cluster’s size may exert an effect on the energy consumption and the load. A cluster, of which includes more cluster members or the distances between cluster members to the CH is greater, may endure heaver load than others with less cluster members or with more compact cluster structure. Thus, an adapted cluster construction is given (lines 5-10 in Algorithm 1). After applying Algorithm 1, the clusters are constructed as shown in Fig 4.

**Step 4 (CH Update)**: When a CH dies in a cluster, it does not mean that this cluster is useless. To balance the energy consumption inside the network, the role of the CH is shifting among all sensor nodes in the same cluster. The CH reselection will be applied in the cluster when the CH find its energy is not enough for maintaining the cluster. The algorithm is similar to the Algorithm 1. The sensor node, which has not been selected to be the CH for a long period has greater chance to be selected as the replacement of the CH. The network lifetime is defined in Formula (11):

$$\begin{array}{c} T=min\{T_{C_{i}}|C_{i}\;is\;a\;cluster\}. \end{array}$$ (11)

### Directional virtual backboard construction

**Fig 5:**
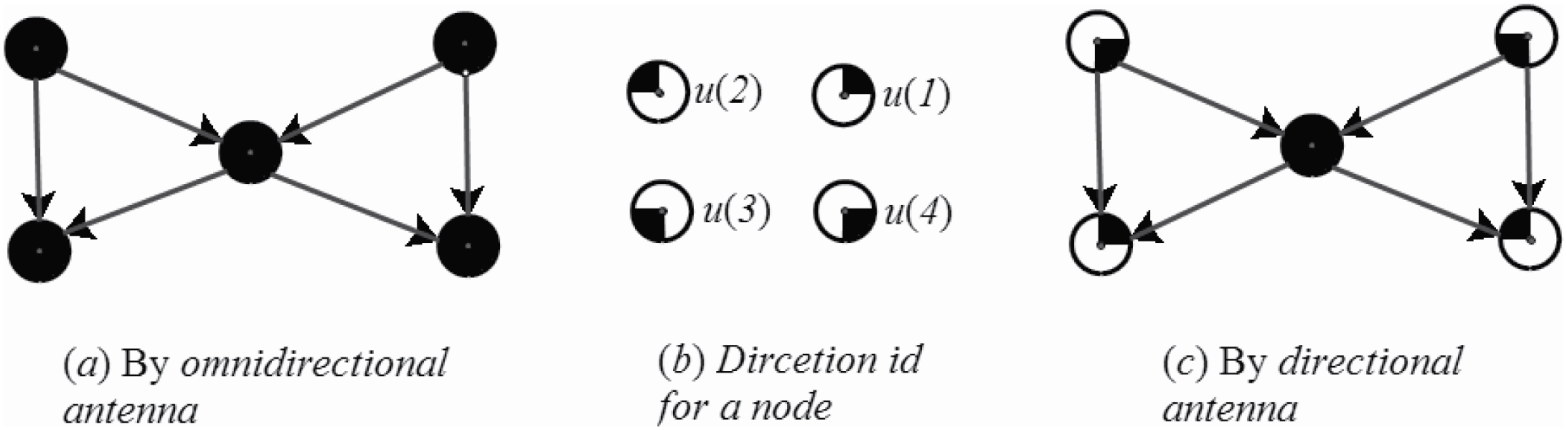
The omni-antenna graph and direction antenna graph.

After the clusters are constructed, the visual data should be transmitted to the sink node by a directional virtual backbone. This section will introduce how to construct an efficient directional virtual backbone between CHs and the sink node. In the omnidirectional antenna environment, sending signals to some directions are unnecessary because there is no receivers on those sectors, which is shown in Fig 5(a). In order to avoid the energy consumption on the unnecessary directions, the directional antennal shown in Fig 5(b) can be used to achieve the goal. By transmitting packets to only the necessary sectors, the WVSN shown in Fig 5(c) can preserve more energy in the transformation progress because the antennas on the unused directions can be turned off.

**Algorithm 2**: Directional virtual backboard construction

1 Each CH searches open the directional antenna *ni*, which is toward the direction of the sink node;

2 **if** (*CH*_*i*_’*s radius* *r*_*i*_ ≤ *r*_*tr*_ *and there is one neighbor* *CH*_*j*_ *exist*) **then**

3  add this link into the edge *E*(*i* → *j*);

4 **end**

5 **else**

6  **if** (*one* *CH*_*i*_’*s radius* *r*_*i*_ ≤ *r*_*tr*_ *and there is no neighbor* *CH* *exist*) **then**

7   expend the radius energy until find one neighbor CH;

8  **end**

9  **else**

10   **while** (*k* < = 4) **do**

11    {close directional antenna *ni*;

12    open the *nj* = ++*ni*(*mod* 4) directional antenna;

13    operate steps 2-8;

14    record the founded parents *CH*_*j*_(*nj*);}

15   **end**

16  **end**

17 **end**

18 compare each *d*(*CH*_*i*_, *CH*_*j*_(*nj*)});

19 record *d*_*min*_(*j*) = *min*{*d*(*CH*_*i*_, *CH*_*j*_(*nj*)})} *j* = 1, 2, 3, 4; then *CH*_*j*_ is *CH*_*i*_’s parents CH;

The constructed directional virtual backbone looks like a directional tree. The sink node is the root of the directional virtual backbone tree *T* at level 0. In the tree, nodes *CH*_*i*_ and *CH*_*j*_ are siblings when they share the same parents. The set of children of *CH*_*i*_ is denoted by *Child*_*i*_. There can be different paths from a cluster head *CH*_*i*_ to the root in different data collection trees of *G*_*CH*_. Algorithm 2 describes the directional virtual backboard construction algorithm in detail. The CHs should find their parents CHs toward the direction of the sink node within the radius (lines 1-8 in Algorithm 2). If CH parents selection is fall in this direction, the suboptimum directional antenna will be selected by opening the directional antennas in turn (lines 10-15 in Algorithm 2). The directional to the sink node and nearest CH (lines 18-19 in Algorithm 2) will be chosen as the parents.

**Fig 6:**
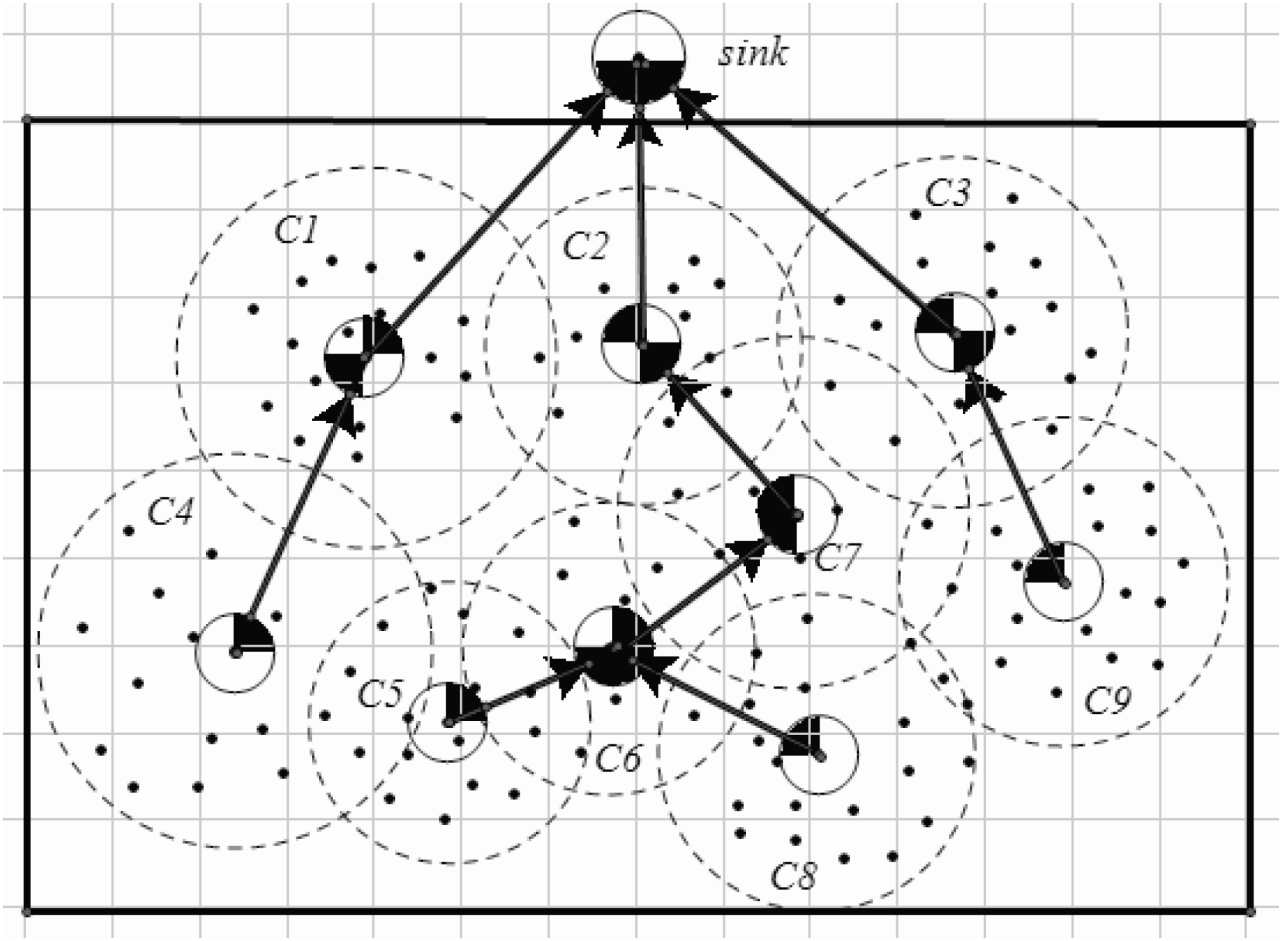
Directional virtual backboard construction.

**Fig 7:**
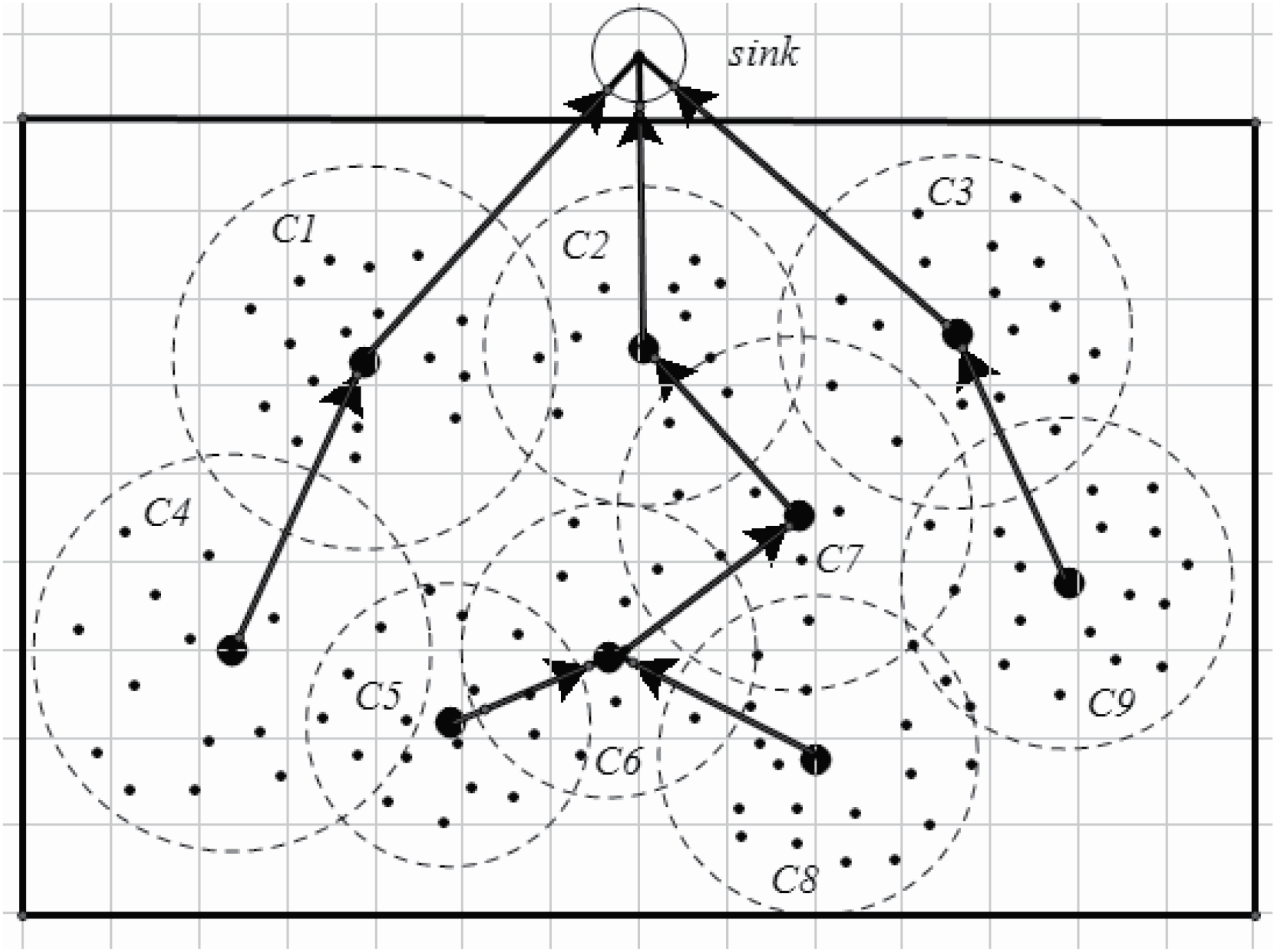
Virtual backboard construction by omni-antenna.

**Fig 8:**
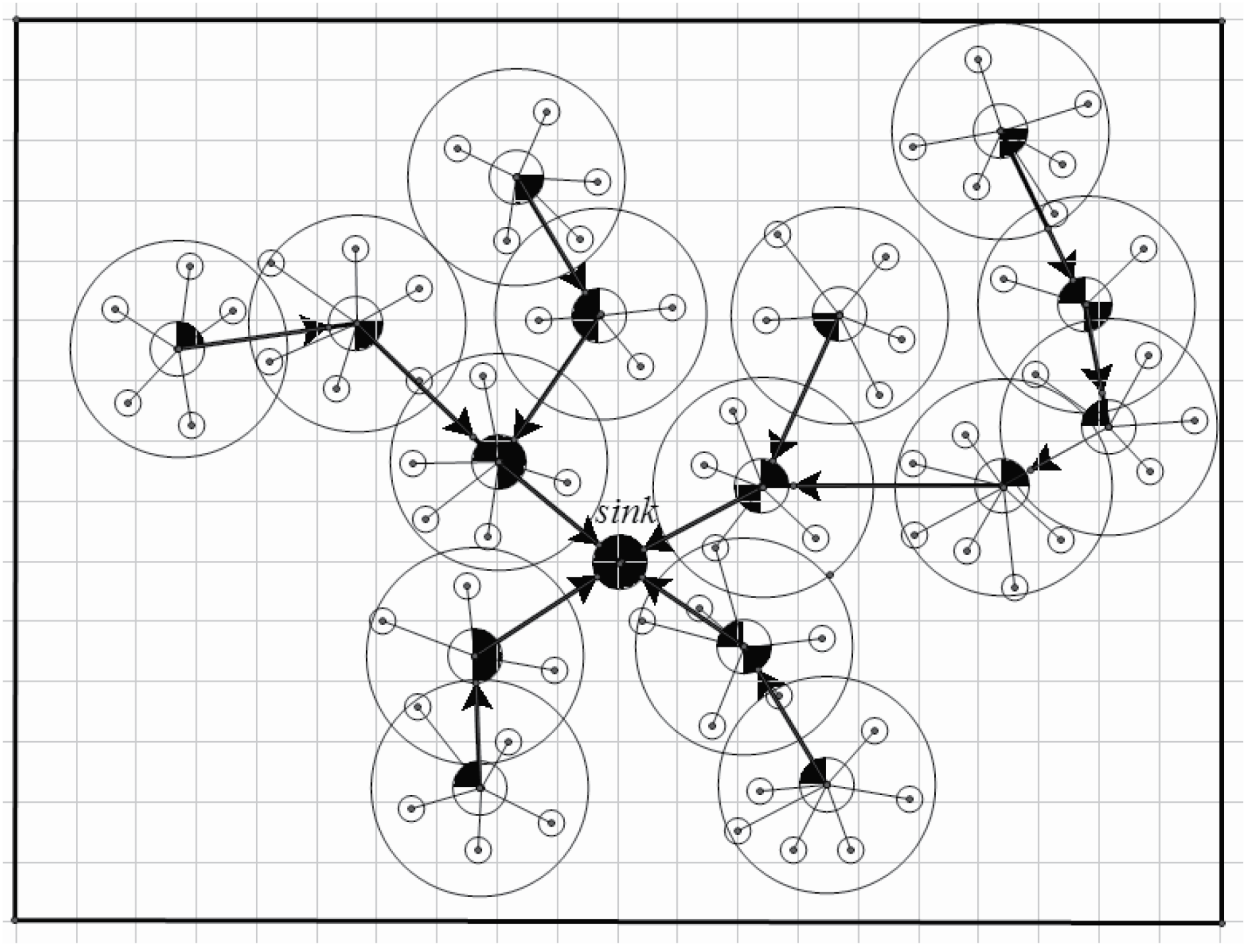
Sink in the center.

An example is shown in Figs 6 (by direction antenna) and Fig 7 (by omni-antenna). It is easy to find that about $\frac{15}{36}$ direction antennas (where 15 is the number of the used directional antennas, and the total number of directional antennas is 9 × 4) are used comparing to the virtual backboard construction by omnidirectional antenna. Without special circumstances, Fig 8 shows another situation, where the sink is in the center. It is easy to find that only 22 direction antennas are used (total number of directional antennas is 15 × 4), almost 60% CH’s energy can be safety comparing to virtual backboard construction by omnidirectional antenna.

### Network coding

The WVSN’s natural property of easy packet loss, which makes it a challenge to transmit information. Network coding is an efficient solution. There are many kinds of network coding algorithms, the random linear network coding and decoding algorithms [29, 31] will be used in this paper. Each source node sends out a data packet of length *n*_0_ symbols. We consider a set of packets $u_{1},u_{2},...,u_{K}\in F_{q}^{1\times n_{0}}$. A coded packet is formed by [*gx*], where $x=\sum_{k=1}^{K}g_{k}u_{k}$ is a linear combination of the data packets and *g* = [*g*_1_, *g*_2_, …, *g*_*K*_] is the global encoding vector for that packet. At the sink, *N* packets are received, which can be represented by Formulas (12) and (13):

$$\begin{array}{c} X={\left[\begin{array}{c} x_{1}\\ .\\ .\\ .\\ x_{K} \end{array}\right]}^{T}=GU\in F_{q}^{K\times n_{0}} \end{array}$$ (12)

$$\begin{array}{c} U={\left[\begin{array}{c} u_{1}\\ .\\ .\\ .\\ u_{K} \end{array}\right]}^{T},G={\left[\begin{array}{c} g_{1}\\ .\\ .\\ .\\ g_{K} \end{array}\right]}^{T}\in F_{q}^{K\times K} \end{array}$$ (13)

The network coding algorithm is applied at coding nodes. In this scheme, the cluster heads are defined as the coding nodes. The network encoding algorithm is described in Algorithm 3. All the necessary linear operations are performed over the finite field *F*_2^8^_ (lines 9-12 in Algorithm 3). The cluster head codes the messages, which are received from its cluster members, into one coded packet. Before this coding progress, the cluster head will judge the data’s repeatability (lines 3-7 in Algorithm 3). Repetitive messages will be discarded.

**Algorithm 3**: network encoding algorithm

1 set a random counting down time *t*;

2 **if** (*some messages are received*) **then**

3  check whether there are same message;

4  **if** (*there are some similar message*) **then**

5   only chose one message from them;

6   set *en* = number of messages;

7  **end**

8 **end**

9 **for** (*j* = 1;*j* < = *en*;*j*++) **do**

10  Select *en* coefficients *C*_*i*_, *i* = 1, 2, …, *en* from *GF*(2^8^) randomly;

11  generates new encoded packets by encoding the *en* data messages *D*_*i*_ given by $X=\sum_{i=1}^{en}C_{i}D_{i}$;

12  broadcast the encoded packets *X*;

13 **end**

The decoding progress starts when the CH receives the encoded packets. If the decoding progress is unsuccessful, some packets need to be retransmitted. Let *RP* refers to the number of encoded packets the considered node need to send out. *RP* = *en* − *rank*(*C*_*n*_), each node sends out a *RP* of encoded packets. We call it the negative *ACK* message. Nodes receiving less than *en* packets can send out *NACK* message to some particular neighbors to retrieve missing data that they need. The nodes that have received *NACK* message send out *RP* encoded packets. This improved encoding algorithm with *NACK* is described as follows.

**Algorithm 4**: network decoding algorithm

1 set *RP* = *en* − *rank*(*C*_*n*_);

2 **if** (*RP* > 0) **then**

3  require another *S* encoded packets;

4  sent *S* NACKs to the child CH;

5 **end**

6 Restart the countdown *t*;

7 receive the packets;

8 decoding the packets;

When the decoding node receives the encoded packets from cluster heads in the upstream, the countdown will begin. If the received packet can be successfully decoded in *t* time slots, the information is combined with their own information, and then forward to the next layer. If the decoding is unsuccessful, the coding node needs to calculate the value of *RP* and send the *RP* *NACKs* to the child CH and restart the countdown when receiving the encoded packet, next time. If it can be decoded within *t* times, then the encoding packages will be forward to the next layer. Otherwise goto this step till the encoding packet can be decoded successful. If the encoding node receives the *NACK* from the next layer cluster head, execution the next stage. The network decoding algorithm is described in Algorithm 4.

## Theoretical analysis

The performances of our scheme will be studied in this section. Derived in Theorem 1, the energy consumption of the proposed method is lower than other methods. The boundary of the network lifetime is given in Theorems 2 and 3.

**Theorem 1**: The energy consumption by the scheme is about $\left(N*\left(1-p_{aver}\right)*\left(\left\lceil \frac{1}{p_{aver}}\right\rceil -1\right)*\left(\frac{a^{2}}{2\pi *N*p_{aver}}*E_{fs}+E_{elec}\right)+N*p_{aver}*{\left(r_{i}\right)}^{2}*E_{fs}*k+E_{elec}*k\right)*pd_{i}$, which is smaller than other omni-directional antennal scheme.

**Proof**: Assume the average transmit radius *r*_*ave*_ is:

$$\begin{array}{c} r_{ave}=\frac{a}{\sqrt{N*p_{aver}\pi }}. \end{array}$$ (14)

where *a* is the length of the network area, *N* is the number of total sensor planted in the network, *p*_*aver*_ is the average probability of the cluster head selection.

Assume *CH*_*i*_ is a cluster head whose average radius is *r*_*i*_, and *ρ*(*CH*_*i*_) is the sensor node distribution density function for the cluster form by node *CH*_*i*_, then the expectation of the distance between cluster member and cluster head can be calculated as:

$$\begin{array}{ccc} E(d^{2}\left(n,j\right)) & = & \int \int_{CH_{i}}*\left(x^{2}+y^{2}\right)*\rho \left(CH_{i}\right)d\;area\left(CH_{i}\right)\\ & = & \int_{0}^{2\pi }\int_{0}^{r_{ave}}r^{3}\frac{Np_{aver}}{a^{2}}drd\theta \\ & = & \frac{a}{2\pi Np_{aver}} \end{array}$$ (15)

This density function is in the trend of the uniform distribution, which is approximately equal to $\frac{Np_{aver}}{a^{2}}$.

The transmit and the receive energy consumption is presented in Formulas (1–3). The total energy consumption is contained by two aspects, the intra-cluster energy consumption and the inter-clusters energy consumption. According to the density function and the consumption model, then the energy consumption of the intra-cluster can be calculated as:

$$\begin{array}{c} E\left(j,n\right)=\sum_{j=1}^{Ne(CH_{i})}d^{2}\left(j,n\right)*E_{fs}+Ne\left(n\right)*E_{elec} \end{array}$$ (16)

where, *Ne*(*CH*_*i*_) is the number of the cluster head *CH*_*i*_’s neighbors. If the nodes are uniformly distributed in the cluster, the expectation of the energy consumption of the intra-cluster is:

$$\begin{array}{ccc} \bar{E(j,n)} & = & N*\left(1-p_{aver}\right)*\left(\left\lceil \frac{1}{p_{aver}}\right\rceil -1\right)\\ & * & \left(\frac{a^{2}}{2\pi Np_{aver}}*E_{fs}+E_{elec}\right) \end{array}$$ (17)

The expectation distance between the CHs and the sink node is:

$$\begin{array}{ccc} E(d\left(CH_{i},sink\right)) & = & \int_{-\frac{a}{2}}^{\frac{a}{2}}\int_{-\frac{a}{2}}^{\frac{a}{2}}\sqrt{x^{2}+y^{2}}*\rho^{2}\left(n\right)dxdy\\ & = & \int_{-\frac{a}{2}}^{\frac{a}{2}}\sqrt{x^{2}+y^{2}}*\frac{1}{a^{2}}dxdy=0.3825a \end{array}$$ (18)

where *ρ*(*CH*_*i*_) is the cluster heads distribution density. Assume the nodes are uniform distribution in the *a* × *a* area. Then the expectation energy consumption by the CHs is:

$$\begin{array}{c} \bar{E(n,s)}=N*p_{aver}*\left[{\left(r_{i}\right)}^{2}*E_{fs}*k+E_{elec}*k\right] \end{array}$$ (19)

If not use multi-hop routing, the expectation energy consumption by the CHs is:

$$\begin{array}{c} \bar{E(n,s)}=N*p_{aver}*\left[{\left(0.3825a\right)}^{2}*E_{fs}*k+E_{elec}*k\right] \end{array}$$ (20)

which is more than our scheme. Then the total energy consumption is:

$$\begin{array}{ccc} & & (N*\left(1-p_{aver}\right)*\left(\left\lceil \frac{1}{p_{aver}}\right\rceil -1\right)*(\frac{a^{2}}{2\pi *N*p_{aver}}*E_{fs}\\ & & \;+E_{elec})+N*p_{aver}*{\left(r_{i}\right)}^{2}*E_{fs}*k+E_{elec}*k)*pd_{i}. \end{array}$$ (21)

where *pd*_*i*_ is the directional probability.

**Theorem 2**: If *VB*_*i*_ is an optimally balanced virtual backbone tree, its lifetime *t*(*VB*_*i*_) is the lifetime of the WVSNs.

**Proof**: Assume that *VB*_*i*_ is an optimally balanced virtual backbone tree and some non-optimal tree *VB*_*j*_ provides the maximum lifetime. According to Formula (7), let *VB*_*j*_ be *σ*_*j*_-balanced and *VB*_*i*_ be *σ*_*i*_-balanced, where *σ*_*i*_ ≥ *σ*_*j*_. Let *CH*_*x*_ lie on the balanced virtual backbone tree *VB*_*i*_ and who has the largest load. According to Definition 10, the network lifetime is *t*(*VB*_*i*_) = *t*(*CH*_*x*_). Since *CH*_*x*_ has the least lifetime in *VB*_*i*_, the load *L*_*CH*_*x*__ of *CH*_*x*_ is the maximum along the virtual backbone tree *VB*_*i*_.

Also, let *CH*_*y*_ have the largest load in *VB*_*j*_. Similarly, we have *t*(*VB*_*i*_) = *t*(*CH*_*y*_). By definition, *L*_*CH*_*y*__ = *σ*_*j*_, *L*_*CH*_*x*__ = *σ*_*i*_. In more balanced virtual backbone tree, each CH’s load is more equivalent, and thus there is *L*_*CH*_*x*__ > *L*_*CH*_*y*__ and *σ*_*i*_ ≥ *σ*_*j*_. Hence, *VB*_*i*_ provides the maximum lifetime for the network.

**Theorem 3**. Based on the DVBDAS scheme, the upper bound network lifetime is:

$$\begin{array}{c} \max T=\frac{E_{0}}{R_{CH_{i}}^{t}E_{Tx}+R_{CH_{i}}^{r}E_{Rx}}\times Np_{aver}. \end{array}$$ (22)

**Proof**: The virtual backbone tree *VB*_*i*_ is active in the interval $\left[\sum_{j=0}^{i-1}t_{j},\sum_{j=0}^{i}t_{j}\right]$, the whole network will die when the energy of one cluster’member are all exhausted. According to Formula (11), the lifetime of a schedule is defined as *T* = *min*{*T*_*C*_*i*__|*C*_*i*_ *is* *the* *cluster*}.

Assume that *E*_0_ is the initial energy for each node. According to Formulas (1–5), the lifetime of *u* can be expressed as:

$$\begin{array}{c} t_{u}=\frac{E_{0}}{R_{CH_{i}}^{t}E_{Tx}+R_{CH_{i}}^{r}E_{Rx}}. \end{array}$$ (23)

Assume that *p*_*aver*_ is the average probability for selecting the CH at each location. The size of the cluster is given as *Np*_*aver*_. The lifetime can be presented as:

$$\begin{array}{c} \max T=\frac{E_{0}}{R_{CH_{i}}^{t}E_{Tx}+R_{CH_{i}}^{r}E_{Rx}}\times Np_{aver}. \end{array}$$ (24)

## Simulation

In this section, we will compare our DVBDAS scheme with the normal routing algorithm without clustering and the scheme based on the cluster with omnidirectional antennas. The data generation rate $R_{i}^{g}$ of every node is calculated in a sensing and reporting cycle. In order to simulate the reality in the real-world scenario, we assume that the probability rate $p_{i}=R_{i}^{g}$ is depended by the area, which fits in a multi-peak Gaussian Distribution. It can be described by Formula (25), which is constructed in our previous work [32]. The simulation results are presented with the average values.

$$\begin{array}{ccc} & & p_{i}=R_{i}^{g}\left(x,y\right)=\frac{{\left(1-x\right)}^{2}}{4}e^{-x^{2}-{\left(y+1\right)}^{2}}\\ & & \;-\frac{5}{6}\left(\frac{x}{5}-x^{3}-y^{5}\right)e^{-x^{2}-y^{2}}\\ & & \;-\frac{1}{36}e^{-{\left(x+1\right)}^{2}-y^{2}}+\frac{1}{2}. \end{array}$$ (25)

### Competition of the energy consumption

**Fig 9:**
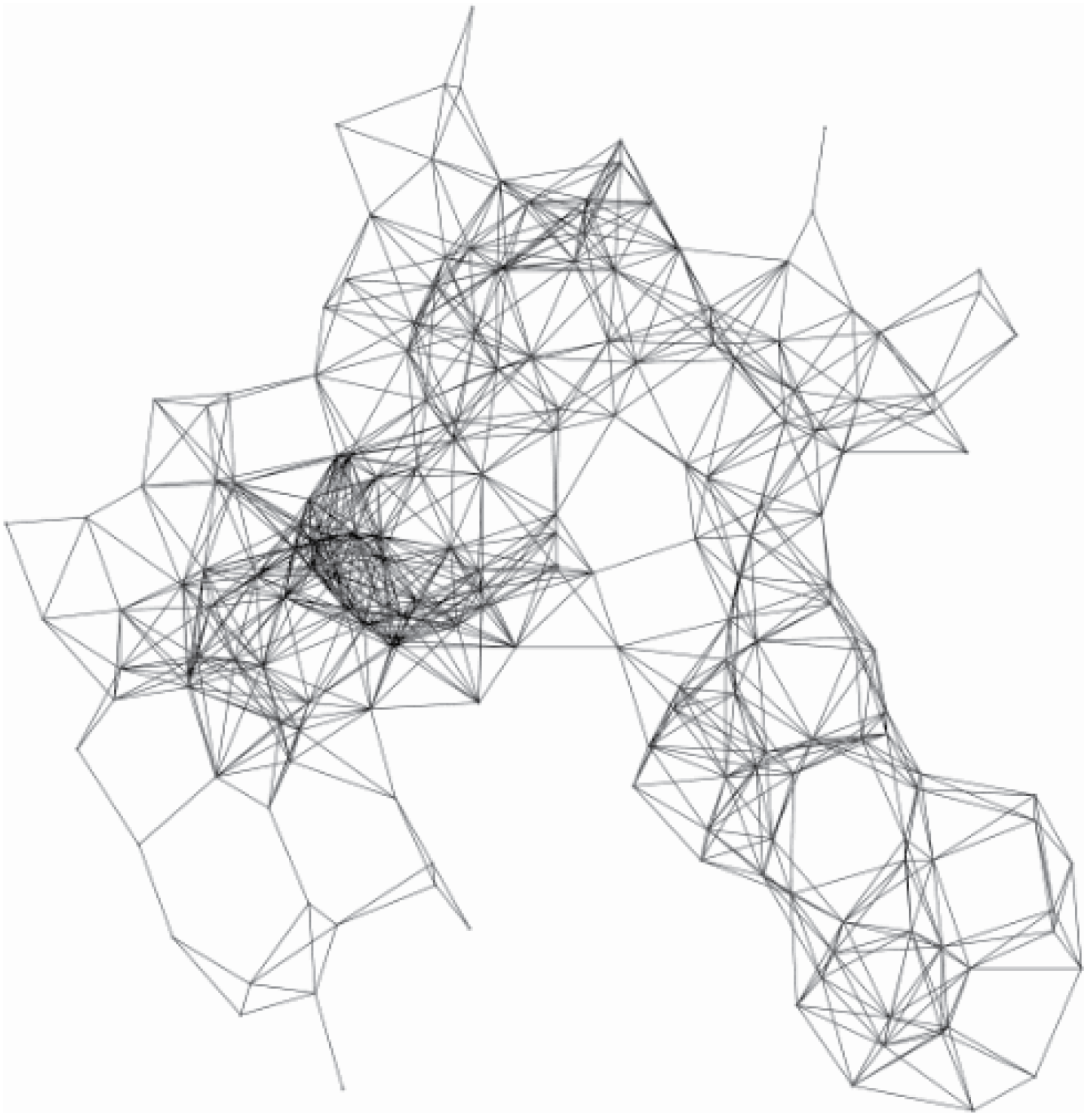
One example of the schemes without cluster.

**Fig 10:**
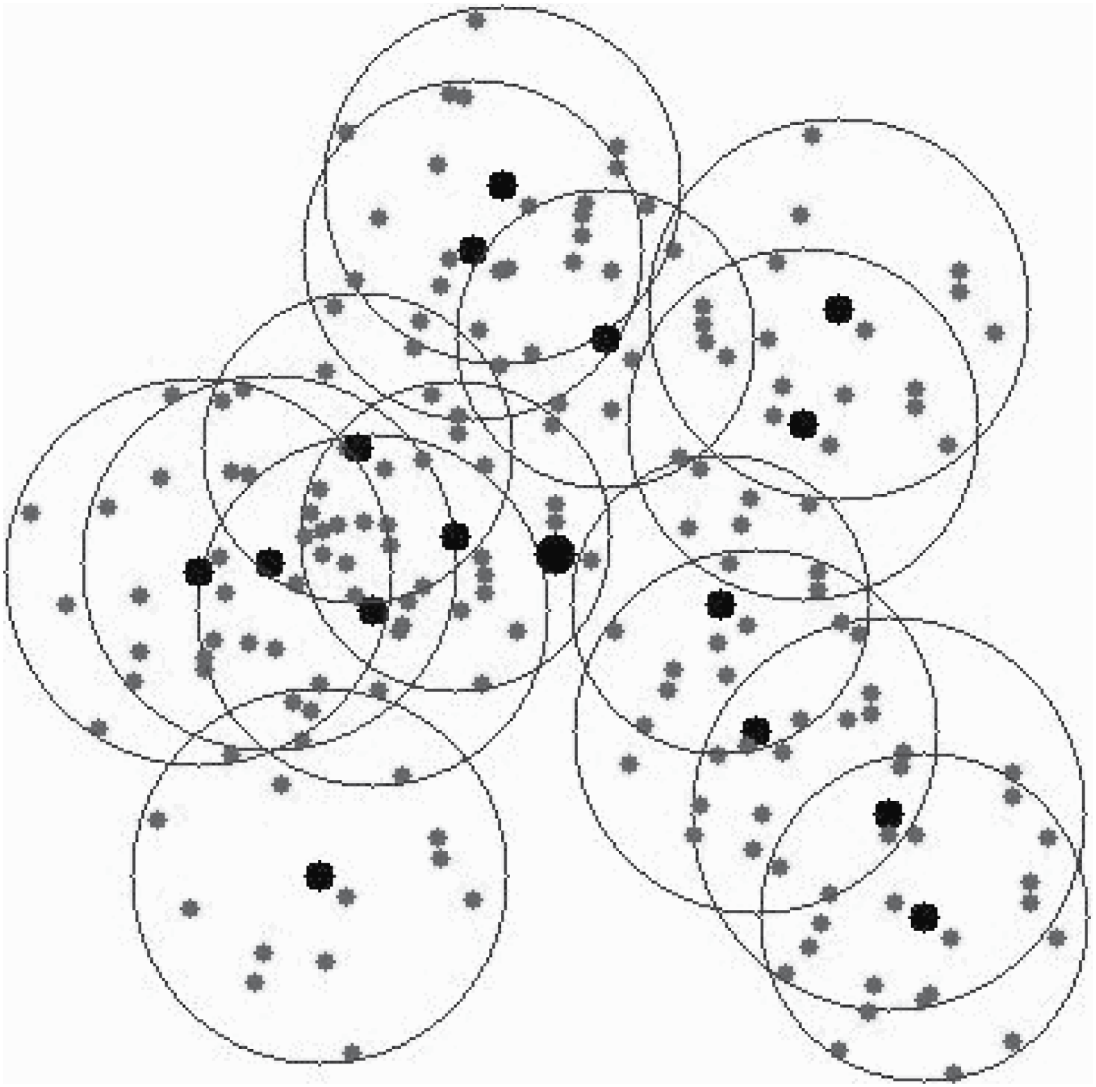
One example of the schemes omni-antenna cluster scheme.

**Fig 11:**
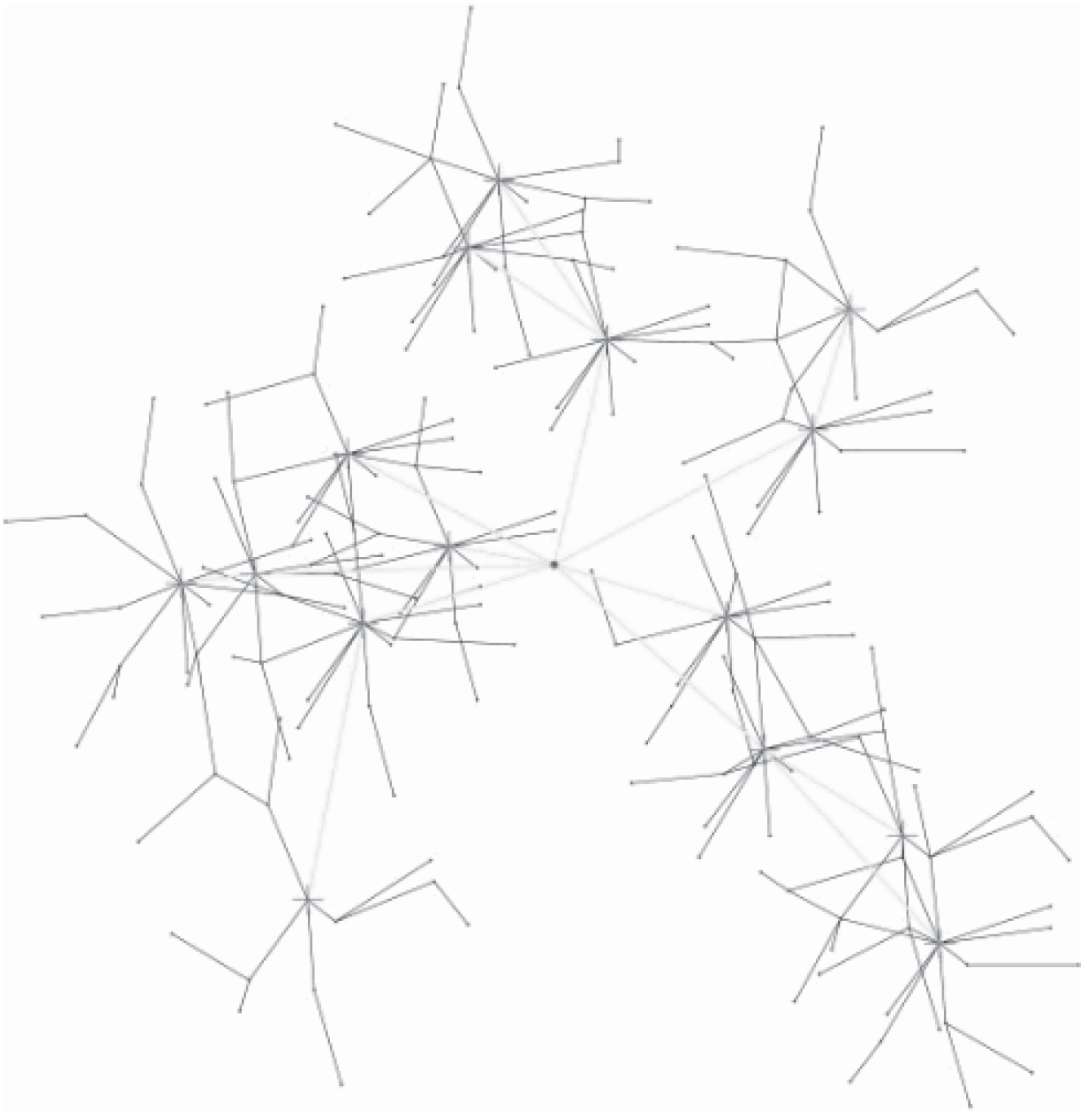
One example of the schemes DVBDAS.

The simulation results are used to evaluate the performance of DVBDAS in the environment built by Visual C++. An example of three schemes is shown in Figs 9–11. About 200 nodes are randomly generated in the 400 × 400 *unit*^2^ network simulation area. The sensing radius of the sensor nodes is set as *r* = 25 *units*. Fig 9 shows the scheme without cluster. The dijkstra algorithm is used in this scheme. Every sensor transmits the messages to the sink node by the shortest path. Fig 10 shows the scheme based on the cluster with the omnidirectional antenna. The CH selection algorithm is the same to our DVBDAS scheme, and the cluster members forward the messages to their cluster head, and then the cluster heads transmit the message to the sink node. Fig 11 shows our DVBDAS scheme.

**Fig 12:**
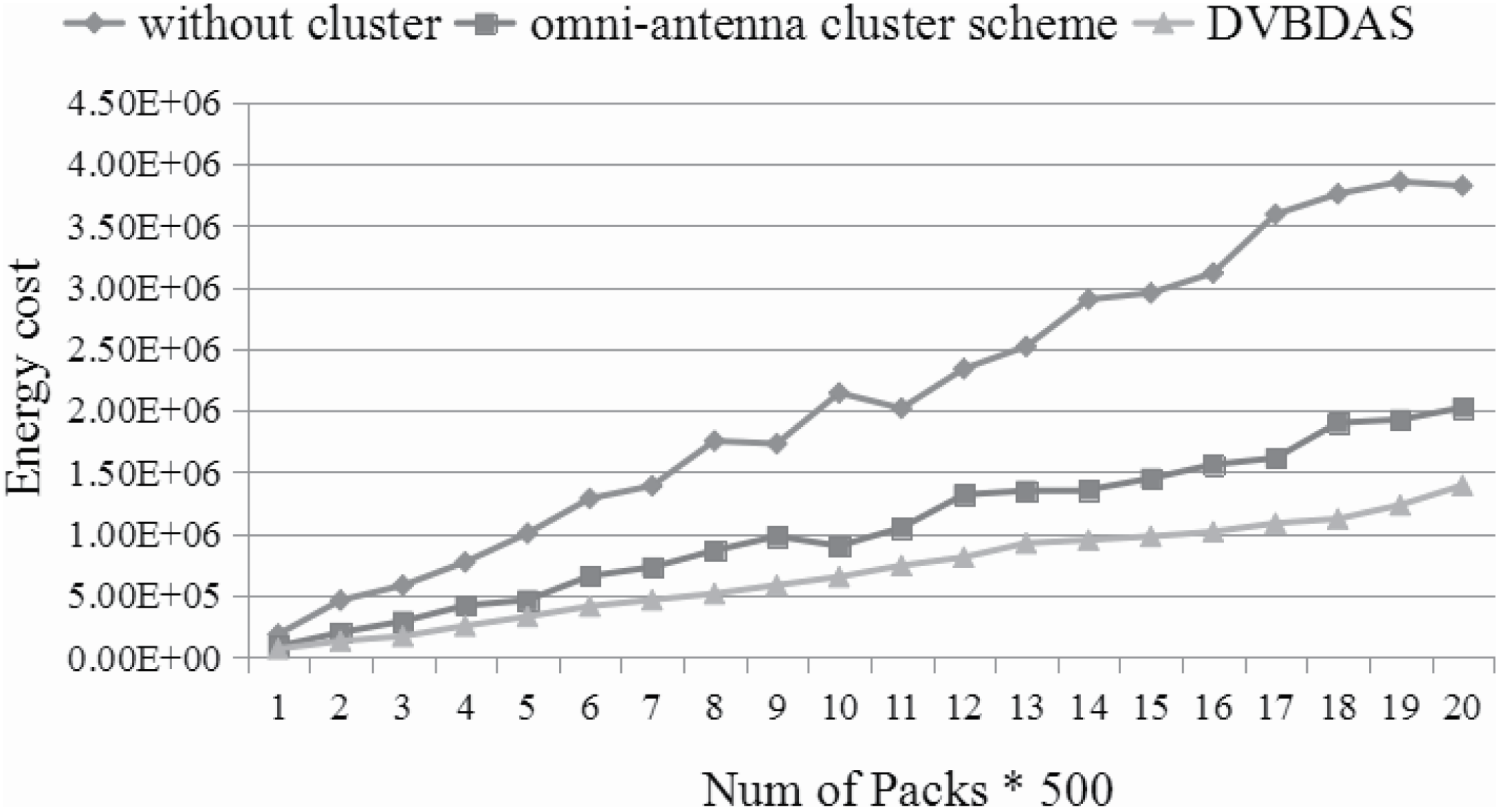
Comparison for energy consumption in 200 sensors.

**Fig 13:**
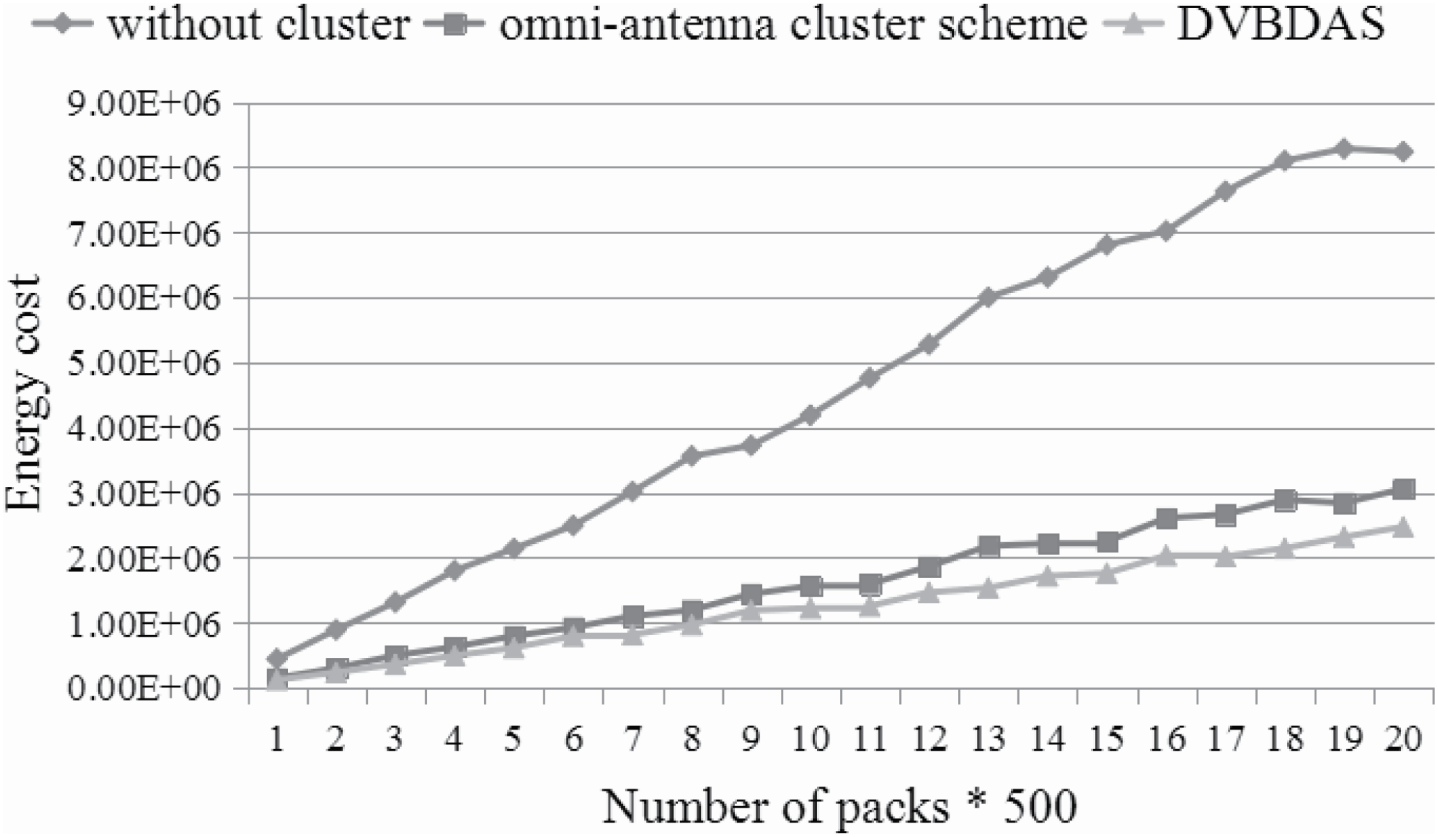
Comparison for energy consumption in 400 sensors.

**Fig 14:**
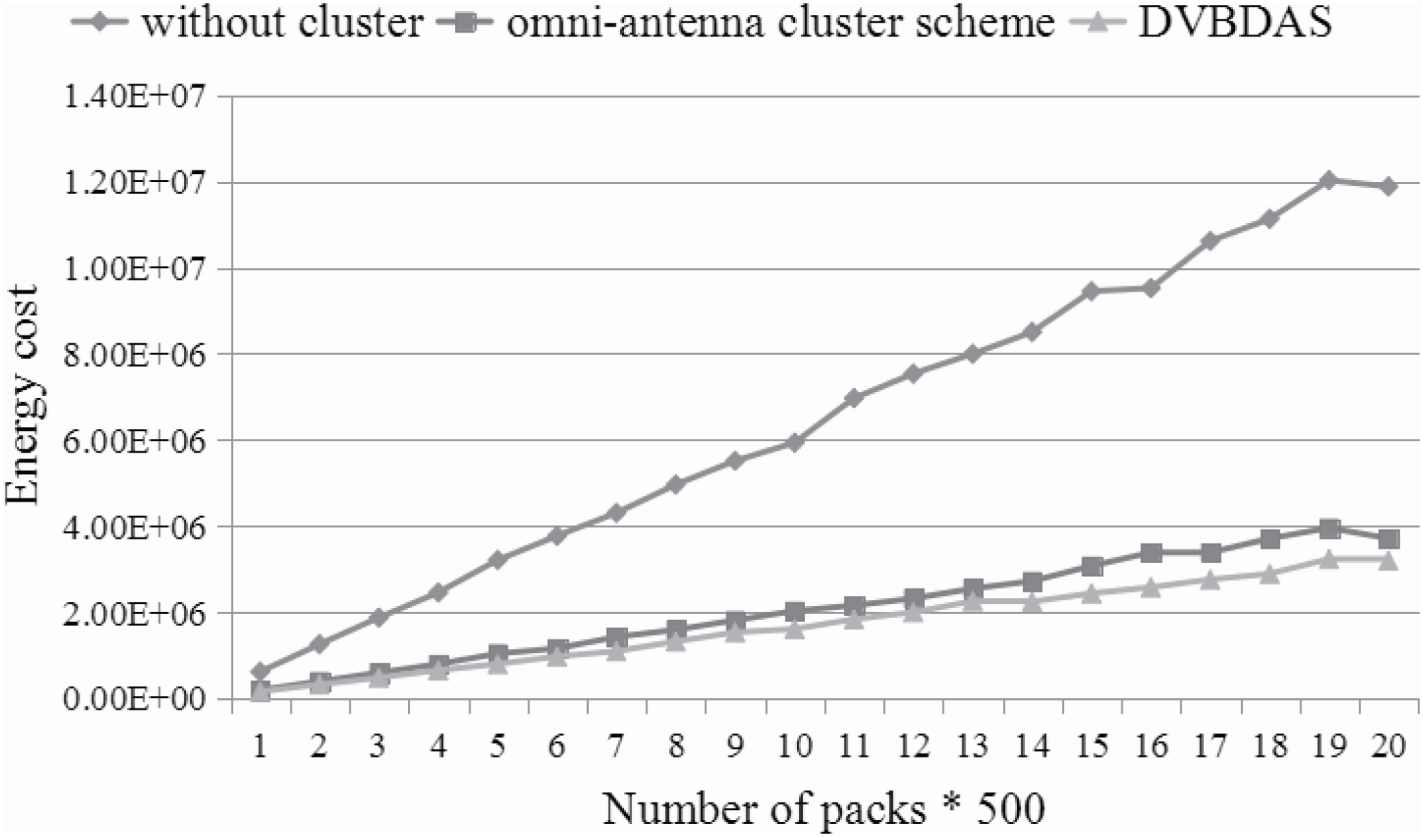
Comparison for energy consumption in 560 sensors.

Figs 12–14 show the energy consumption based on three kind of schemes. The numbers are averaged over 50 repeats. In every repeat, the WVSN is randomly assigned to handle 500 − 10000 packets from the terminal nodes to the sink node. Since there are many similar packets, we assume each sensor transmits the packets with the probability rate, which is introduced in Formula (25). Furthermore, in order to analyze more behaviors in different situations, the number of the sensor can be changed. In this simulation, Figs 12–14 show the comparison between energy consumptions with 200, 400 and 560 sensors, respectively. According to the simulation results, the energy consumption of WVSN with different schemes is quite different. Fig 12 shows that the energy preservation improves about 60% and 35% by our DVBDAS scheme, competing to the scheme without cluster and the scheme based on the cluster of the omnidirectional antenna, respectively. The improvements of the energy preservation are about 70% and 22% (see Fig 13), 73% and 20% (see Fig 14), respectively. The lager the number of sensors, the higher the effectiveness is achieved. The reason is that more packets can be transmitted to the cluster head and being combined into one encoded packet.

### Competition of the lifetime

This section will provide the comparison over three schemes in terms of the network lifetime, which is calculated by the messages transmitted to the sink node, successfully. For the scheme without cluster, since the sink node’s neighbors take more loads, the messages can not reach the sink node when the sink node’s neighborhoods are all dead. The lifetime of the network ends when all neighbors of the sink node are dead. The lifetime of the cluster based scheme ends when there is no CH can be selected in a cluster for transmitting the messages.

**Fig 15:**
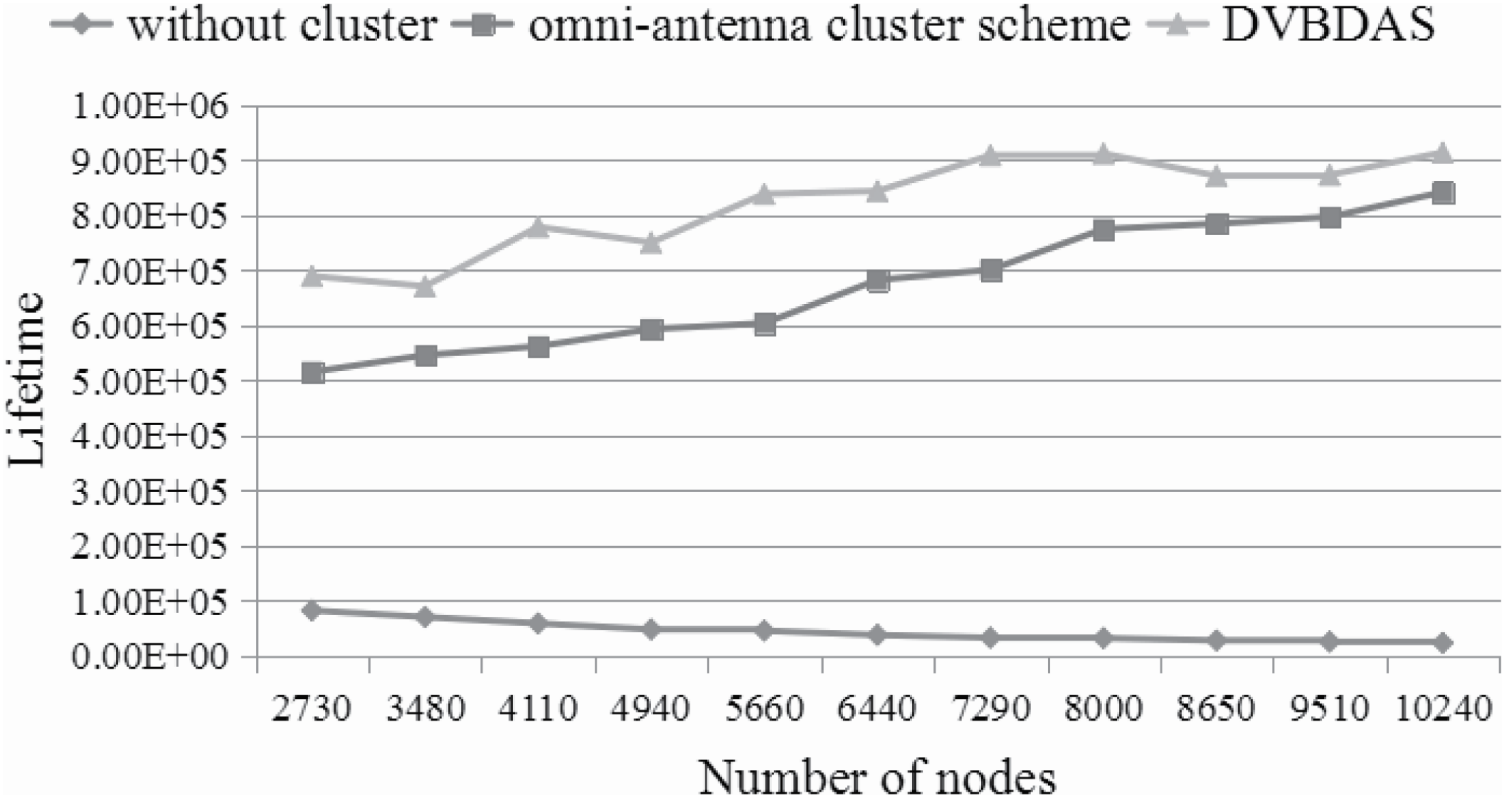
The comparison of lifetime.

Sensors are also deployed in a 400*400 *unit*^2^ area. The sensing range is fixed to 25. The transmit range is adjustable. The initial node battery capacity is 2500 mAh, the transmission power is 60mW, the receiving power is 45mW. The number of nodes is risen from 2730 to 10240. Fig 15 shows the lifetime of different schemes in such case. The lifetime obtained by the scheme without cluster is the shortest. For example, when the number of nodes is 2730, the lifetime is only 82738.6s. At the same time, the lifetimes are 515318s and 689105s for the cluster-based scheme with the omnidirectional antenna and our proposed DVBDAS scheme, respectively. When the number of nodes is 10240, the lifetime is only 23888.4s for the scheme without cluster, the lifetimes are 841366s and 913236s for the cluster-based scheme with the omnidirectional antenna and DVBDAS scheme, respectively. It is easy to understand that the lifetime obtained by the proposed DVBDAS algorithm is the longest, and the lifetime without cluster is declined by the increase of the number of the sensors, while the cluster based schemes are increased on the contrary. Furthermore, compared with the Fig 14, the energy preservation is not large enough when the number of sensors in the network is larger, because the CMs require more energy to forward packets to the CHs. However, the lifetime for our proposed DVBDAS scheme is greater, according to the directional antenna used in CHs’ forwarding process, which is consist with the analysis given in section 4.2. According to the analysis, the lifetime of the DVBDAS can be improved near 25% comparing to the scheme based on the cluster with the omnidirectional antenna, which is also consist with Theorem 3.

## Conclusion

Data gathering is a fundamental task in WVSNs. Directional antennas have a number of advantages over omnidirectional antennas. By focusing energy only in the intended direction, directional antenna can increase the potential for spatial reuse and can provide longer transmission/reception ranges by consuming the same amount of energy. The feature of the directional antennas and the visual data make WVSNs more complex. Therefore, in this paper, we focus on constructing a Directional Virtual Backbone based Data Aggregation Scheme (DVBDAS), which can obtain the minimum energy consumption and maximum network lifetime by combining the cluster algorithm, the directional virtual backbone construction algorithm and the network coding mechanism. Finally, the performances of our proposed DVBDAS are studied by the theoretical analysis and the simulations. The results indicate that the energy consumption by the proposed DVBDAS is smaller than other schemes but the network lifetime is longer than others.

Tracking the moving target is considered as one of the important applications in WVSNs. To the best of our knowledge, improving the tracking quality and extending the network lifespan are two main objects for the target tracking, which are usually contradictory due to limited energy of sensor nodes in WVSNs. However, the lifetime and the tracking quality also are two contradictory issues. In the future, we will focus on finding the balance of these two significate problems.

## Supporting information

S1 Fig One example of the schemes without cluster. (PDF)

S2 Fig One example of the schemes without cluster omni-antenna cluster scheme. (PDF)

S3 Fig One example of the schemes without cluster DVBDAS. (PDF)

S1 File Data. Relevant data underlying the findings described in manuscript.
(ZIP)
