# Supplementary figures and images for: Directional virtual backbone based data aggregation scheme for Wireless Visual Sensor Networks
Source: PLoS One. 2018 May 15;13(5):e0196705. doi: 10.1371/journal.pone.0196705 (PMC5953460; doi:10.1371/journal.pone.0196705)

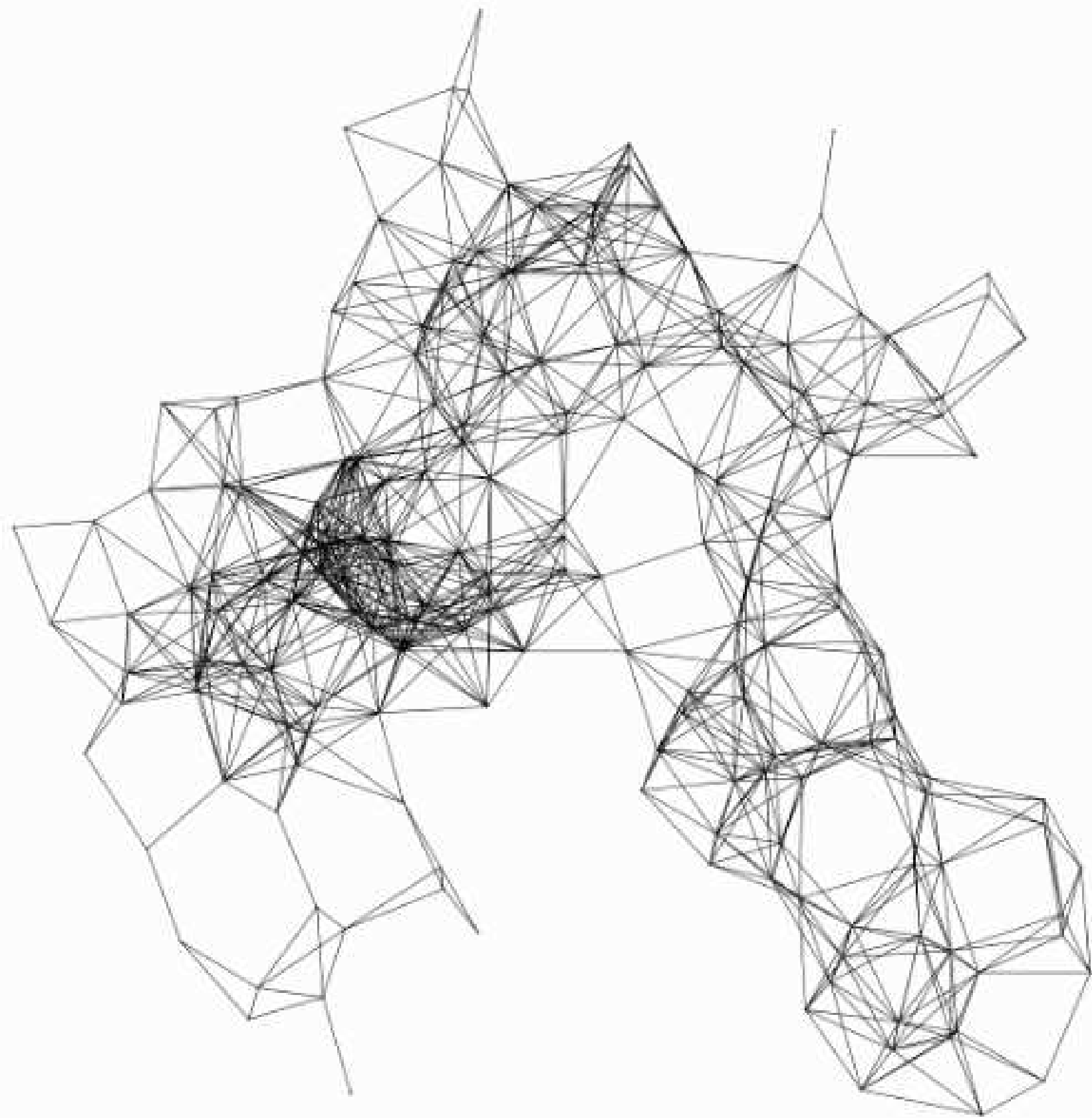

Supplement: S1 Fig — (PDF) [file pone.0196705.s001.pdf]

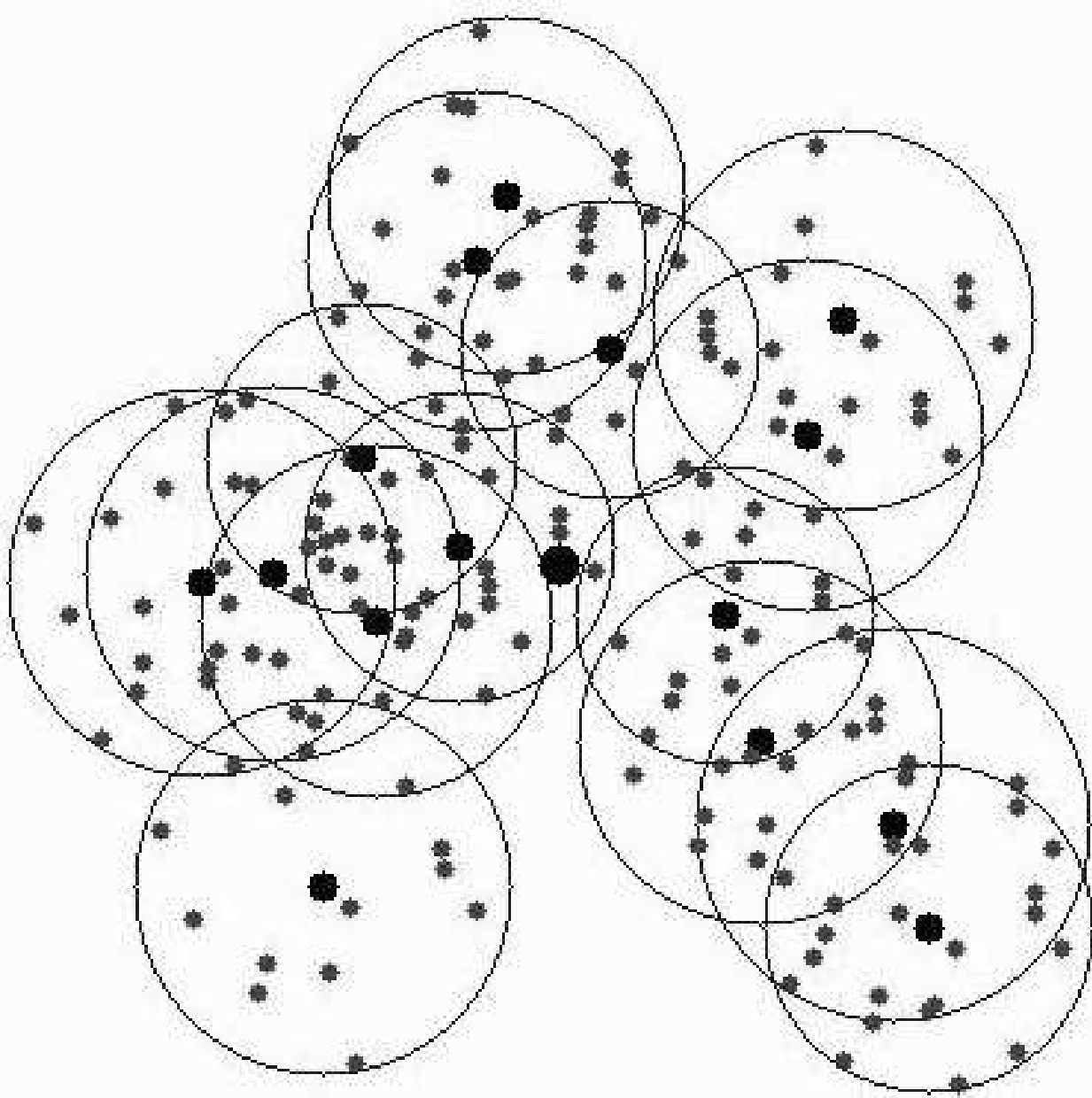

Supplement: S2 Fig — (PDF) [file pone.0196705.s002.pdf]

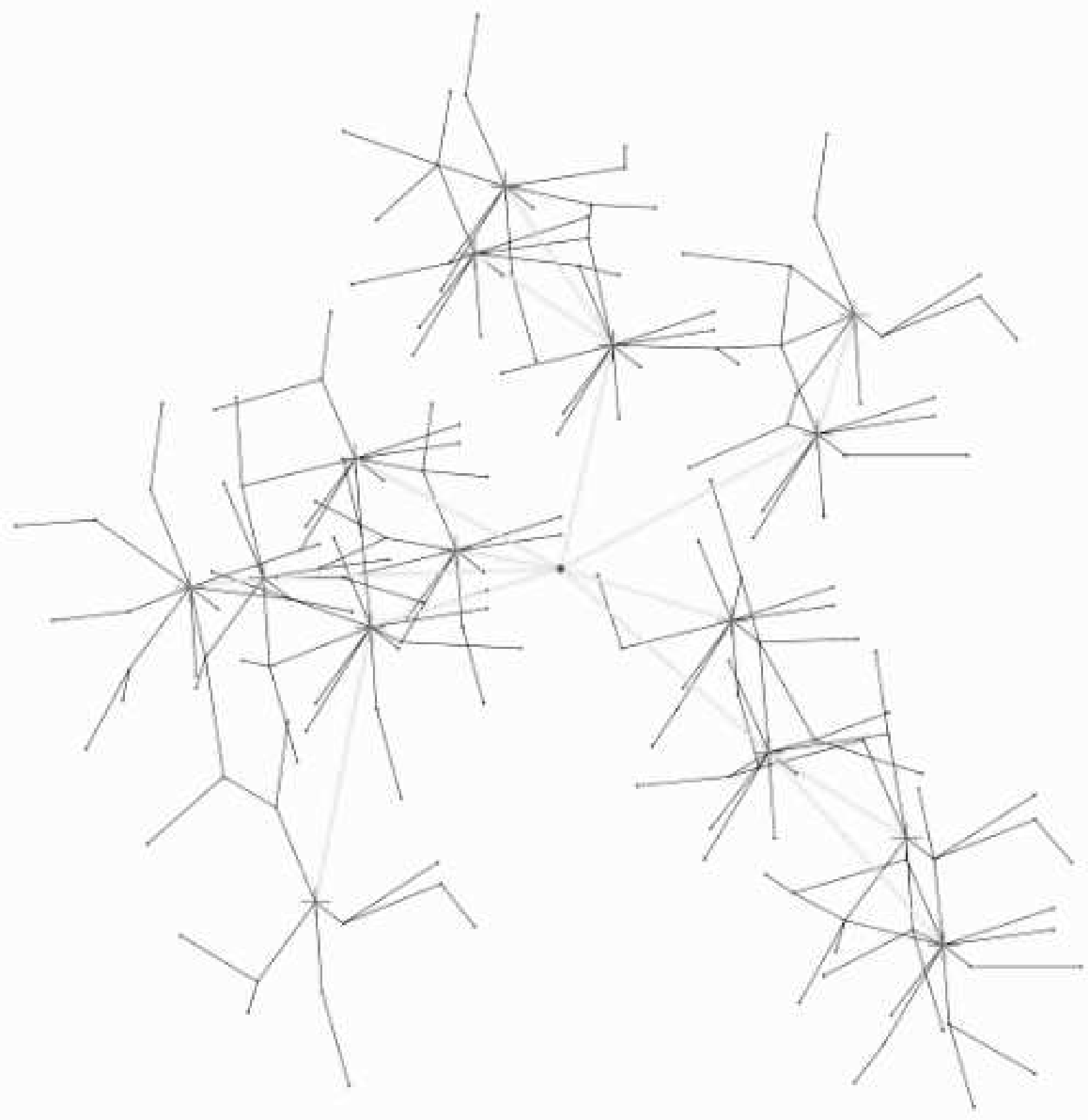

Supplement: S3 Fig — (PDF) [file pone.0196705.s003.pdf]
